# Mesenchymal stem cells ameliorate inflammation and pyroptosis in diabetic cardiomyopathy via the miRNA-223-3p/NLRP3 pathway

**DOI:** 10.1186/s13098-024-01389-7

**Published:** 2024-07-02

**Authors:** Qu Yang, Qi Chen, Sihui Li, Jun Luo

**Affiliations:** 1 Department of Rehabilitation Medicine, The 2nd Affiliated Hospital, Jiangxi Medical College, Nanchang University, Nanchang, Jiangxi 330006 China; 2 Jiangxi Medical College, Nanchang University, Nanchang, Jiangxi 330006 China

**Keywords:** Diabetic cardiomyopathy, Mesenchymal stem cell therapy, Pyroptosis, Inflammation, microRNA

## Abstract

**Background:**

Diabetic cardiomyopathy (DCM) stands as the primary cause of heart failure and mortality among patients with diabetes. Nevertheless, conventional treatment approaches are limited in their ability to effectively prevent myocardial tissue damage itself. Mesenchymal stem cell (MSC) therapy exhibits immense potential for treating DCM; however, the precise mechanisms involved in regulating inflammatory responses and pyroptosis processes, an emerging form of cellular death, within myocardial cells remain elusive. Hence, it is imperative to further elucidate the precise underlying mechanisms to facilitate the clinical implementation of MSC therapy.

**Methods:**

In vivo, we established a DCM mouse model by administering streptozotocin and fed the mice a high-glucose and high-fat diet, followed by MSC therapy. Cardiac function and myocardial injury were evaluated through echocardiography and histological analysis. Furthermore, the levels of inflammation and pyroptosis were assessed using ELISA, Western blotting, and qRT-PCR. In vitro experiments involved inducing H9C2 myocardial cell damage with high glucose treatment, followed by coculture with MSCs to investigate their role in modulating inflammation and pyroptosis mechanisms.

**Results:**

MSCs can maintain cardiac function and alleviate myocardial injury in mice with DCM. Moreover, they effectively suppress the activation of NLRP3 and reduce the release of inflammatory factors (such as IL-1β and ROS), thereby further downregulating the expression of pyroptosis-related proteins including NLRP3, Caspase-1, and GSDMD. Additionally, we experimentally validated that MSCs exert their therapeutic effects by promoting the expression of miR-223-3p in cardiac myocytes; however, this effect can be reversed by an miR-223-3p inhibitor.

**Conclusion:**

MSCs effectively mitigate the release of inflammatory factors and cell lysis caused by pyroptosis through the regulation of the miR-223-3p/NLRP3 pathway, thereby safeguarding cardiomyocytes against damage in DCM. This mechanism establishes a novel theoretical foundation for the clinical treatment of cardiac conditions utilizing MSCs.

**Supplementary Information:**

The online version contains supplementary material available at 10.1186/s13098-024-01389-7.

## Introduction

Diabetic cardiomyopathy (DCM) has emerged as a significant cause of mortality among individuals with diabetes, representing a grave complication of the disease [1]. The pathological manifestation of DCM is progressive worsening of heart function leading to end-stage heart failure [2]. Currently, conventional treatment for DCM primarily focuses on blood glucose control and uses medication intervention to delay the deterioration of cardiac function [3]. Although these classical treatments have been used in clinical practice, they fail to prevent the progression of myocardial tissue lesions [4, 5]. Therefore, the development of therapeutic strategies targeting the underlying pathogenic mechanisms of DCM is highly important. It is widely acknowledged that mesenchymal stem cells (MSCs) exhibit remarkable efficacy in organ repair while demonstrating minimal adverse effects [6–8]. The present study revealed a novel mechanism through which MSCs exert their therapeutic effects on cardiomyocytes via microRNA, thereby complementing the existing understanding of how MSCs effectively treat DCM.

In recent years, the therapeutic and preventive potential of MSCs in the context of DCM has gradually gained recognition alongside advancements in stem cell research and regenerative medicine [7, 8]. MSCs possess robust self-renewal capabilities, immune regulatory properties, and pluripotent differentiation capacities, rendering them widely applicable for cellular transplantation investigations due to their low immunogenicity [9, 10]. Numerous studies have demonstrated that MSCs can mitigate myocardial injuries resulting from diverse factors through their immunomodulatory, anti-inflammatory, antioxidant stress-relieving, and antiapoptotic effects [11–13]. MSCs play a crucial role in inhibiting cell pyroptosis following cardiac and cerebral injuries induced by events such as cardiopulmonary resuscitation (CPR), acute kidney injury (AKI), and traumatic brain injury (TBI), among others [14–16]. However, despite these findings in other contexts, the specific involvement of MSCs in DCM remains understudied, and the underlying mechanism is unclear.

Pyroptosis, a type of programmed cell death associated with an inflammatory process, has emerged as a novel regulatory mechanism in cardiovascular diseases [17, 18]. It induces cell lysis by perforating the cell membrane and releasing proinflammatory factors. The occurrence of pyroptosis is closely linked to the activation of the nucleotide-binding oligomerization domain, leucine-rich repeat and pyrin domain-containing protein 3 (NLRP3) inflammasome through various stimuli [19, 20]. This cascade facilitates the conversion of pro-caspase-1 to caspase-1 and transforms pro-interleukin-1β (pro-IL-1β) and pro-IL-18 into mature IL-1β and IL-18. Additionally, activated caspase-1 cleaves gasdermin D (GSDMD), generating a GSDMD-N-terminal that can puncture cell membranes and promote the release of inflammasomes and cytokines. Consequently, this process contributes to collagen deposition and fibrotic formation, thereby exacerbating the severity of DCM [21–23]. Therefore, investigating the targeted impact of MSCs on the downregulation of the NLRP3 inflammasome could offer a novel avenue for exploring the potential of MSCs to inhibit myocardial pyroptosis in DCM.

MicroRNAs (miRNAs) are a highly conserved group of noncoding small RNAs that exert significant influence on posttranscriptional gene expression regulation and various biological processes [24–26]. Recent studies have demonstrated that the beneficial effects of MSCs may be attributed, in part, to their ability to regulate host-derived miRNAs, subsequently modulating organ-specific gene expression [27, 28]. In T pallidum-infected endothelial cells and renal inflammation, miR-223-3p has been shown to negatively regulate NLRP3 expression and suppress inflammasome activation and pyroptosis, with both nucleotide and ‘seed’ sequences for miR-223-3p and NLRP3 being 100% homologous between mice and humans [29–31]. However, it remains unclear whether MSCs mediate the alleviation of myocardial pyroptosis and inflammation through the modulation of miR-223-3p.

In this study, in vitro and in vivo functional genomics investigations demonstrated the potential of MSC therapy to alleviate myocardial injury in DCM. This therapeutic effect is achieved by enhancing miR-223-3p expression in cardiomyocytes, leading to the inhibition of NLRP3-mediated pyroptosis and inflammation. These findings provide a novel theoretical basis for utilizing MSCs as a treatment strategy for myocardial injury in DCM.

## Materials and methods

### Induction of the DCM mouse model

C57BL/6J mice (6 weeks old, 18–20 g) were randomly selected to construct the DCM model, and the remaining mice in the normal group were used as controls for subsequent experiments. The mice were continuously fed a high-glucose and high-fat diet, while the normal group was fed a normal diet. After 4 weeks, these mice were induced by a single intraperitoneal (i.p.) injection of streptozotocin (STZ, 60 mg/kg, Sigma, USA) in fresh 0.1 M citrate buffer (pH 4.5), while normal mice were injected with an equal volume of citrate buffer. Then, mice were fed a high-glucose and high-fat diet for 3 months. In the DCM model group, mice with blood glucose levels of ≥ 16.6 mM were classified as diabetic mice and randomly divided into the MSC and DCM groups. The MSC group was treated with human bone MSCs, while the DCM group was not. All protocols were approved by the Laboratory Animal Science Center of Nanchang University.

### Preparation and administration of MSCs

Human bone MSCs were acquired from the American Type Culture Collection (ATCC, Manassas, United States) and cultured in Dulbecco’s modified Eagle’s medium (DMEM) supplemented with 10% fetal bovine serum (FBS) and 1% penicillin streptomycin at 37℃ and 5% CO_2_. Cells with good growth status were selected for subsequent experiments. MSCs were injected into the MSC group mice via tail vein three times in total, at the 16th, 18th, and 20th weeks, with a resuspension of 2 × 10^6^ MSCs in 0.2 mL of sterile saline each time. The normal and DCM groups were injected with 0.2 mL of sterile normal saline as a control.

### Echocardiography

Transthoracic echocardiography was performed four weeks after the last treatment to assess cardiac function. After anesthetizing the mice with isoflurane, two-dimensional targeted M-mode traces were obtained at the papillary muscle level using an echocardiography system (Vevo 770, VisualSonics, Canada). Strict tests were carried out on the left ventricular end-diastolic internal diameter (LVIDD), left ventricular end-systolic internal diameters (LVIDS), left ventricular ejection fraction (LVEF), fractional shortening (FS), and early to late diastolic mitral annular velocity (E’/A’) through the system.

### Evaluation of myocardial injury

At the end of the experiment (week 24), the mice were euthanized and their heart weight to body weight ratio (HW/BW) was determined to assess myocardial hypertrophy. Blood samples were collected to quantify the cardiac injury markers creatine kinase (CK) and creatine kinase-MB (CK-MB) using an automatic biochemical analyzer.

All heart tissues were isolated and fixed with 4% paraformaldehyde, embedded in paraffin, and sliced for further use. Hematoxylin-eosin (HE) staining was used to observe changes in histology and pathology. Masson’s trichrome staining was used to evaluate the accumulation of collagen fibers. The expression of specific proteins was detected by immunohistochemical staining. The sections were incubated with primary antibodies at room temperature for 60 min, followed by incubation with secondary antibodies, against NLRP3 (1: 50, Proteintech, China), Caspase-1 (1: 100, Proteintech), GSDMD (1: 50, Proteintech), Collagen-I (1: 100, Proteintech) and Collagen-III (1: 100, Proteintech). Sections were then stained with diaminobenzidine (brown) and hematoxylin (blue).

### Culturing and treatment of H9C2 myocardial cells

H9C2 myocardial cells were treated in vitro with a high glucose (HG) medium to induce cardiomyocyte injury [11]. Cells were inoculated in 6-well plates and cultured in DMEM supplemented with normal glucose (5 mmol/L) for 6 h. Then, MSCs were cocultured with H9C2 cells at a ratio of 1:2 (MSC: H9C2) for 48 h in a Transwell system, and the medium was replaced with fresh HG (33 mmol/L) medium containing either a negative control (NC) inhibitor or a miR-223-3p inhibitor. Before coculture, MSCs were seeded into the upper chamber of a Transwell culture plate for 24 h. Cells were divided into four groups: (1) the control (normal glucose) + NC group, (2) the HG + NC group, (3) the HG + MSCs + NC group, and (4) the HG + MSCs + miR-223-3p inhibitor group.

### Cell viability and survival analysis

​The viability of H9C2 cells in the above groups was detected using a cell counting kit-8 (CCK-8, Solarbio, Beijing, China) assay. After 48 h of treatment, cells were collected and seeded into 96-well plates (approximately 5000 cells/well) and cultured with 100 µL of medium for 6 h. Then, 10 µL of CCK-8 working reagent was added and incubated for 2 h at 37℃. The optical density (OD) was detected at 450 nm using a microplate reader to indicate cell viability.

Cell survival in each group after treatment was detected using a Hoechst 33342/PI staining kit (Solarbio, Beijing). Cells grown in a 6-well plate were washed twice with phosphate-buffered saline and then stained with Hoechst 33342 and propidium iodide (PI) dye solution for 20–30 min in the dark according to the manufacturer’s instructions. Images were observed using a fluorescence microscope. PI-negative cells were considered viable cells, and PI-positive cells were considered dead cells.

### Enzyme-linked immunosorbent assay (ELISA)

An ELISA kit (Jingmeibio, Jiangsu, China) was used to detect inflammation levels in the myocardium and H9C2 cells. Heart tissues were homogenized in cold saline at a ratio of 1:9. Afterward, the samples were centrifuged at 3000 rpm for 10 min at 4℃, and the supernatant was collected for subsequent analysis. The supernatant of the H9C2 cells in each group was collected. All samples were subjected to ELISA analysis, following the manufacturer’s protocol, to determine the levels of interleukin-1β (IL-1β) and reactive oxygen species (ROS).

### Dual-luciferase reporter gene assay

H9C2 cells were seeded into 6-well plates at a density of 70–80% and cotransfected with the luciferase plasmids psiCHECK2-NLRP3-WT or psiCHECK2-NLRP3-Mut and miRNA-NC or miR-223-3p mimics (100 nM) using Lipofectamine^®^ RNAiMAX Reagent. After 48 h of transfection, the cells were harvested to detect firefly and renilla luciferase activities using the Dual-Luciferase Reporter Assay System (Promega). The results are presented as the ratio of the two luciferase activities.

### Western blot assays

Total proteins were extracted from heart tissue and cells by sodium dodecyl-sulfate polyacrylamide gel electrophoresis and then transferred to polyvinylidene difluoride membranes. The membranes were then incubated overnight at 4 °C with the following primary antibodies: anti-NLRP3 (1: 500, Proteintech), anti-Caspase-1 (1: 500, Proteintech), anti-GSDMD (1: 500, ABclonal, China), anti-IL-1β (1: 300, Proteintech), and anti-GAPDH (1: 5000, Proteintech). The following day, the membrane was incubated with the corresponding secondary antibodies for 1 h. After washing with Tris-buffered saline containing Tween-20, proteins were visualized using an enhanced chemiluminescence (ECL) Kit, and chemical luminescence reactions were detected using a Bio-Rad luminescence imaging system.

### Quantitative real-time PCR (qRT-PCR)

Total RNA was extracted from tissues and cells with TRIzol reagent (Invitrogen, United States). cDNAs were synthesized using a reverse transcription kit (Takara, Japan), and then amplified using SYBR Green Master Mix (Takara). mRNA expression levels were detected using a Real-time ABI 7900 HT System (Applied Biosystems, CA, United States). The primer sequences for the genes were as follows: NLRP3, F: 5’-TAAGAACTGTCATAGGGTCAAAACG-3’, R: 5’- GTCTGGAAGAACAGGCAACATG-3’; GAPDH, F: 5’-CCTCGTCCCGTAGACAAAATG-3’, R: 5’-TGAGGTCAATGAAGGGGTCGT-3’; miR-223-3p, F: 5’-ACACTCCAGCTGGGTGTCAGTTTGTCAAAT-3’, R: 5’-TGGTGTCGTGGAGTCG-3’; U6, F: 5’-CTCGCTTCGGCAGCACA-3’, R: 5’-AACGCTTCACGAATTTGCGT-3’. GAPDH and U6 were used as internal controls. Three independent experiments were performed for each reaction in triplicate. The results of the experiment were calculated using the 2^−ΔΔCT^ method.

### Statistical analysis

All the data are presented as the mean ± standard deviation (SD) and were analyzed using GraphPad Prism. Differences between the two groups were analyzed using Student’s t-test. Differences between multiple groups were analyzed using analysis of variance (ANOVA). *P* < 0.05 was considered to indicate statistical significance. All experiments were repeated at least three times.

## Results

### MSCs maintain cardiac function and alleviate myocardial injury in DCM mice

**Fig. 1:**
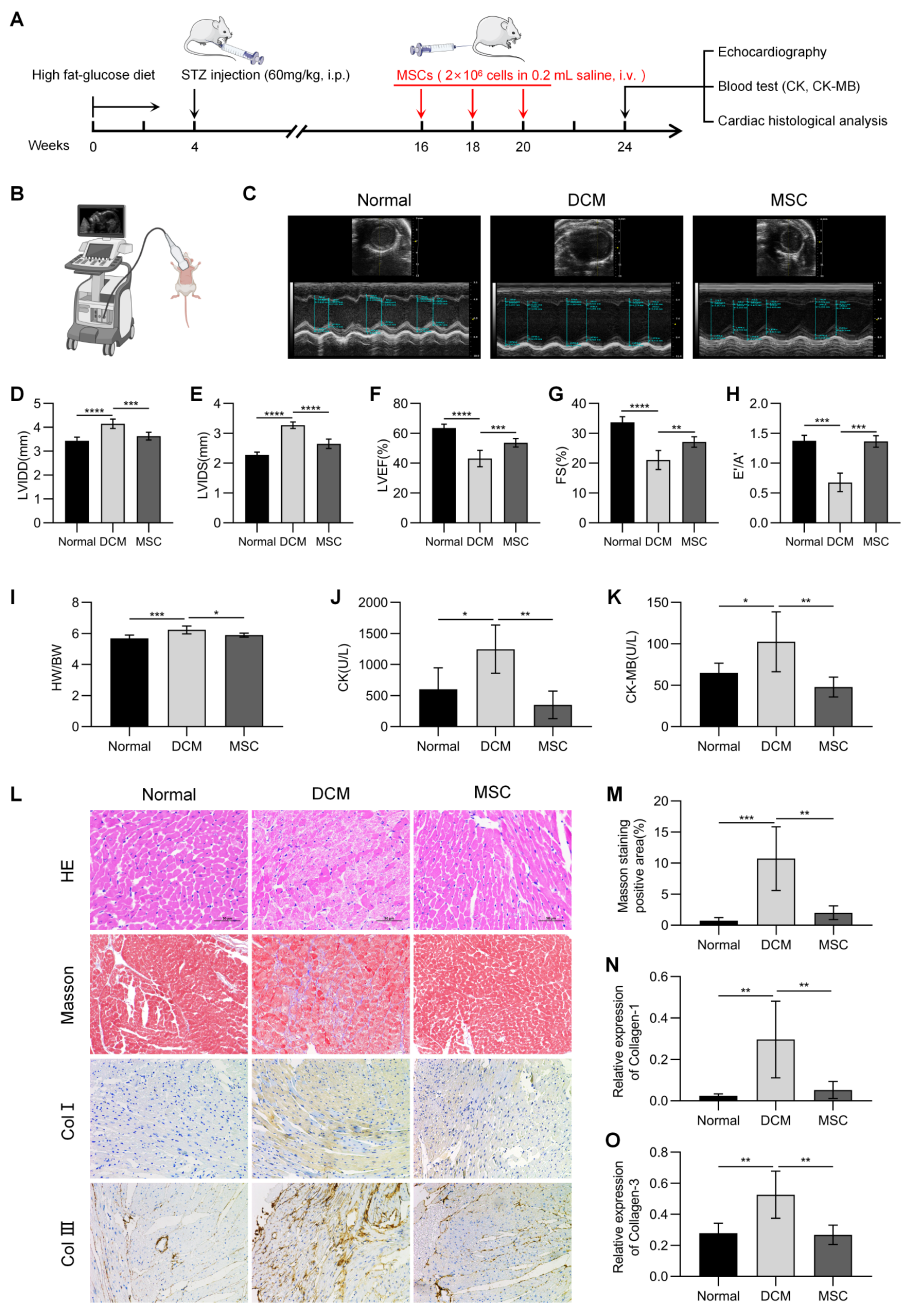
MSCs maintain cardiac function and alleviate myocardial injury in DCM mice. (**A**) The flowchart illustrates the establishment of a DCM mouse model and subsequent treatment with MSCs. (**B, C**) Mouse cardiac function was evaluated through echocardiography. (**D-H**) The results of LVIDD, LVIDS, LVEF, FS, and E’/A’ were recorded to assess cardiac function (*n* = 6). (**I**) Cardiac hypertrophy was evaluated by calculating the ratio of heart weight to body weight (HW/BW). (**J, K**) Quantification of the cardiac injury markers creatine kinase (CK) and creatine kinase-MB (CK-MB). (**L-O**) HE staining, Masson’s trichrome staining, and immunohistochemical staining for type I collagen and type III collagen were performed on myocardial samples, followed by quantitative analysis. (Normal group: a normal diet; DCM group: a high-glucose and high-fat diet; MSC group: a high-glucose and high-fat diet + MSC infusion. *P < 0.05, **P < 0.01, ***P < 0.001, ****P < 0.0001)

C57BL/6J mice were fed a high-glucose and high-fat diet in combination with STZ injections to establish a DCM model, followed by caudal vein injection of MSCs to treat myocardial injury (Fig. 1A). The effects of MSC administration on cardiac diastolic and systolic functions in DCM mice were evaluated by echocardiography (Fig. 1B and C). The results showed that the LVIDD and LVIDS of mice in the DCM group were markedly greater than those in the normal group, and those of mice in the MSC group were significantly less than those in the DCM group (Fig. 1D and E). The LVEF and FS of the DCM group were reduced compared with those of the normal group and improved in the MSC group compared with those of the DCM group (Fig. 1F and G). The E′/A′ ratio was significantly lower in the DCM group than in the normal group, significantly greater in the MSC group than in the DCM group, and similar to that in the normal group (Fig. 1H). In addition, the HW/BW ratio was greater in the DCM group than in the normal and MSC groups, but it was similar between the MSC and normal groups (Fig. 1I).

We collected blood samples from the mice to measure CK and CK-MB levels, which reflect the extent of myocardial injury. As shown in Fig. 1J and K, the CK and CK-MB levels in the DCM group were significantly increased, while those in the MSC group were as low as those in the normal group. Heart samples from all mice were collected for histological examination to further analyze myocardial injury. HE staining revealed that the myocardium was hypertrophic and disordered in the DCM group, while it was well-preserved in the MSC group (Fig. 1L). Masson’s trichrome staining revealed a significant increase in the deposition of blue collagen in the myocardium of the DCM group (Fig. 1L and M). The degree of myocardial fibrosis was evaluated by staining for type 1 collagen and type 3 collagen. As shown in Fig. 1L and M, and 1O, the expression of type I collagen and type III collagen in the DCM group was elevated compared to that in the normal group, while the expression in the MSC group was comparable to that in the normal group. The above results indicate that injecting MSCs into the circulatory system can reduce myocardial injury in diabetic mice, maintain cardiac function and myocardial structure, and prevent the occurrence of cardiomyopathy.

### MSCs mitigate NLRP3-mediated inflammation and pyroptosis in DCM mice

**Fig. 2:**
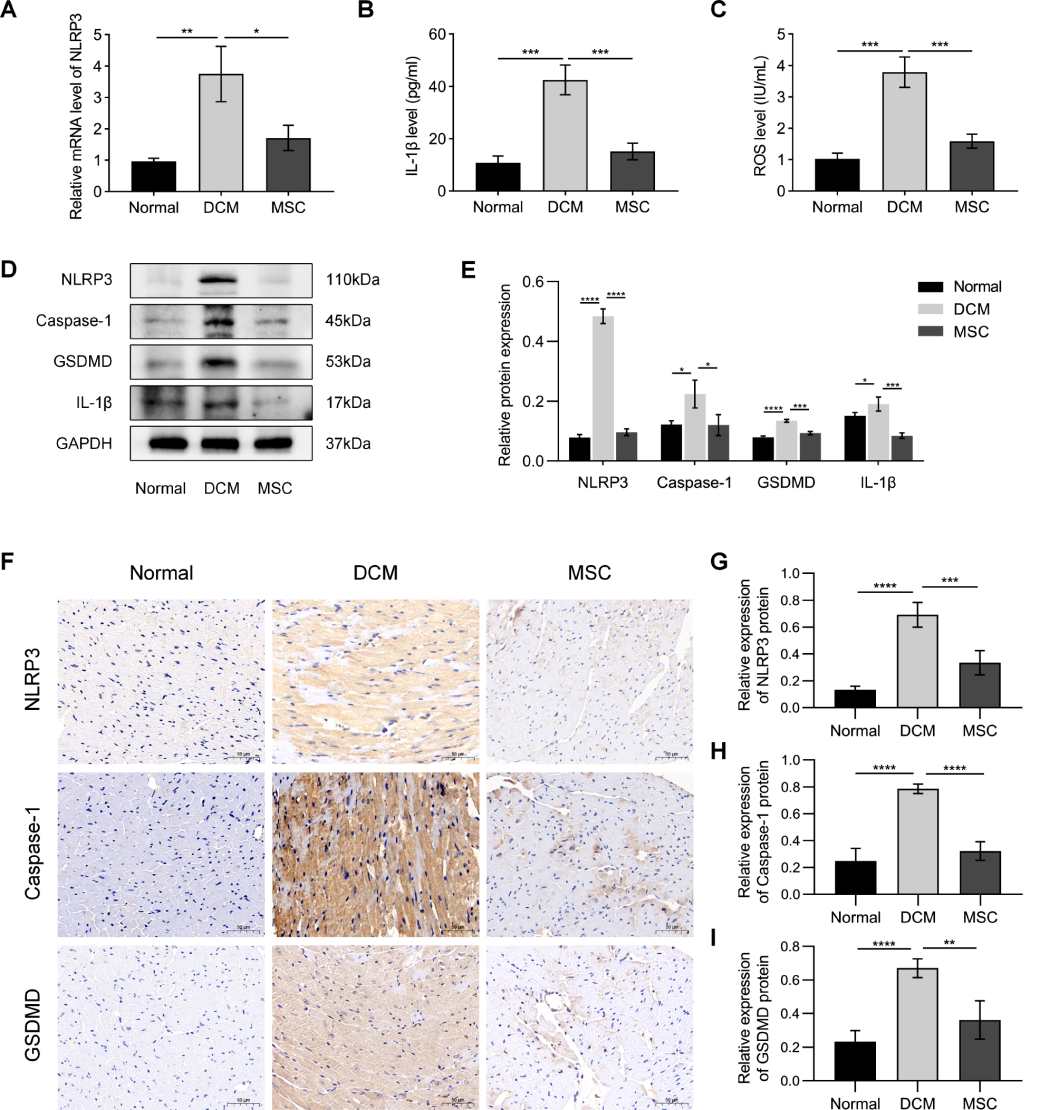
MSCs mitigate NLRP3-mediated inflammation and pyroptosis in DCM mice. (**A**) MSC treatment decreased the expression levels of NLRP3 in the myocardium. (**B-C**) The levels of the proinflammatory cytokine IL-1β and reactive oxygen species (ROS) in myocardial tissue were measured using ELISA. (**D, E**) The protein levels of NLRP3, Caspase-1, GSDMD and IL-1β in myocardial tissue were measured and quantitatively analyzed. (**F-I**) Immunohistochemical staining of the NLRP3, Caspase-1, and GSDMD proteins in myocardial tissue was performed, followed by quantitative analysis

Due to its crucial role in regulating inflammation and pyroptosis, NLRP3 plays a vital role in the progression of DCM. The qRT-PCR results demonstrated a significant upregulation of NLRP3 mRNA expression in the DCM group compared to the normal group, whereas a notable downregulation was observed in the MSC group relative to the DCM group (Fig. 2A). The levels of IL-1β and ROS, which are indicators of inflammation, were elevated in the DCM group but not in the MSC group (Fig. 2B and C). On the other hand, Western blot assays revealed significant upregulation of the NLRP3, Caspase-1, GSDMD, and IL-1β proteins in the DCM group compared to the normal group. In contrast, these protein expression levels were significantly lower in the MSC group than in the DCM group (Fig. 2D and E). Similarly, immunohistochemical analysis revealed significantly increased protein expression of NLRP3, Caspase-1, and GSDMD in the DCM group compared to both the normal and MSC groups (Fig. 2F and I). The data presented herein demonstrate the efficacy of MSCs in mitigating myocardial inflammation and pyroptosis in DCM mice through their regulatory influence on NLRP3.

### MiR-223-3p specifically targets and modulates the expression of NLRP3 in H9C2 cells

**Fig. 3:**
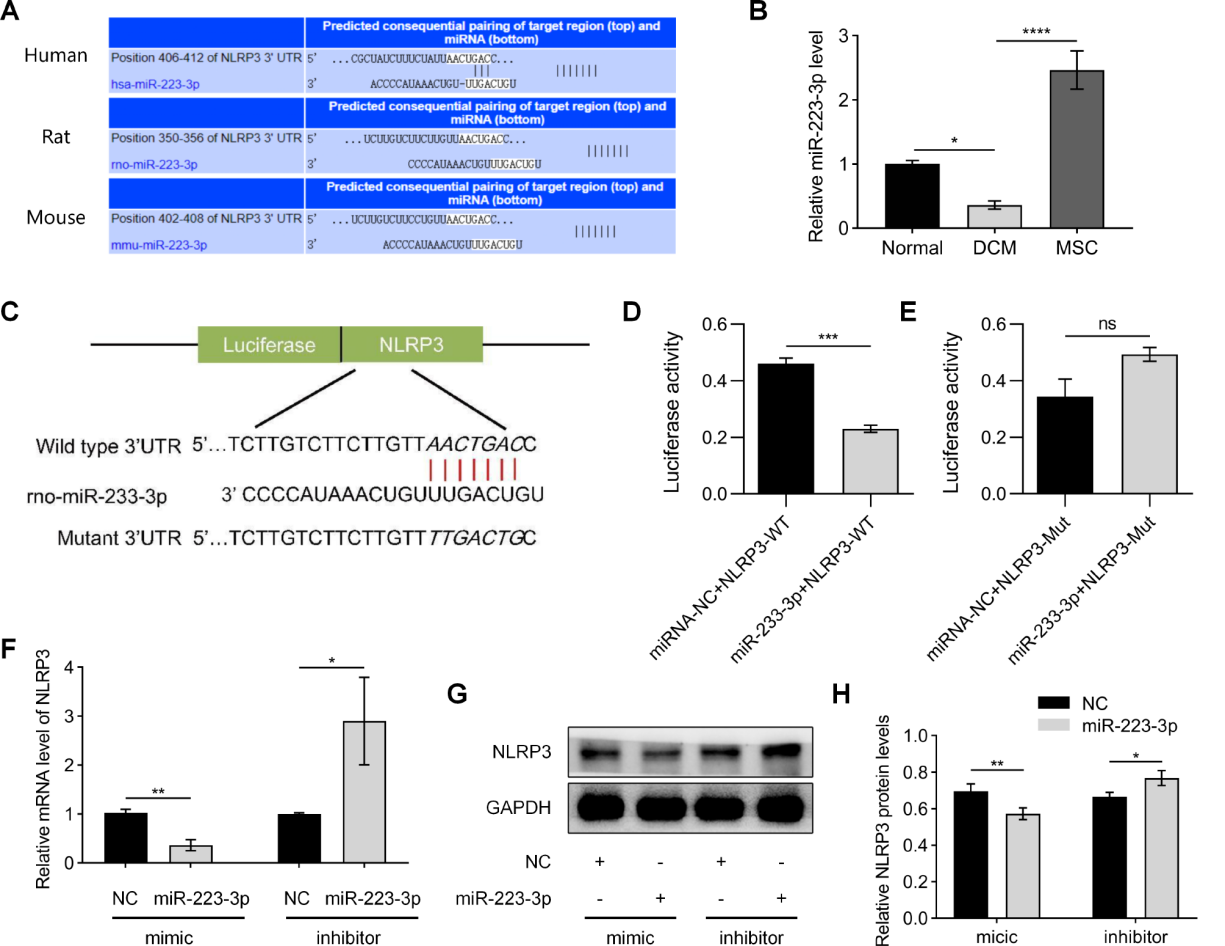
MiR-223-3p specifically targets and modulates the expression of NLRP3 in H9C2 cells. (**A**) The bioinformatics analysis software TargetScan (www.targetscan.org) was used to identify the specific binding site of miR-223-3p within the 3’-UTR of NLRP3. (**B**) The expression of miR-223-3p in myocardial tissue was detected using qRT-PCR. (**C-E**) Structural validation of the interaction between miR-223-3p and NLRP3 was performed in H9C2 cells using a dual-luciferase reporter gene assay. (**F-H**) Functional validation of the miR-223-3p/NLRP3 interaction was conducted by transfecting mimics or inhibitors into H9C2 cells. (^ns^*P* > 0.05)

**Fig. 4:**
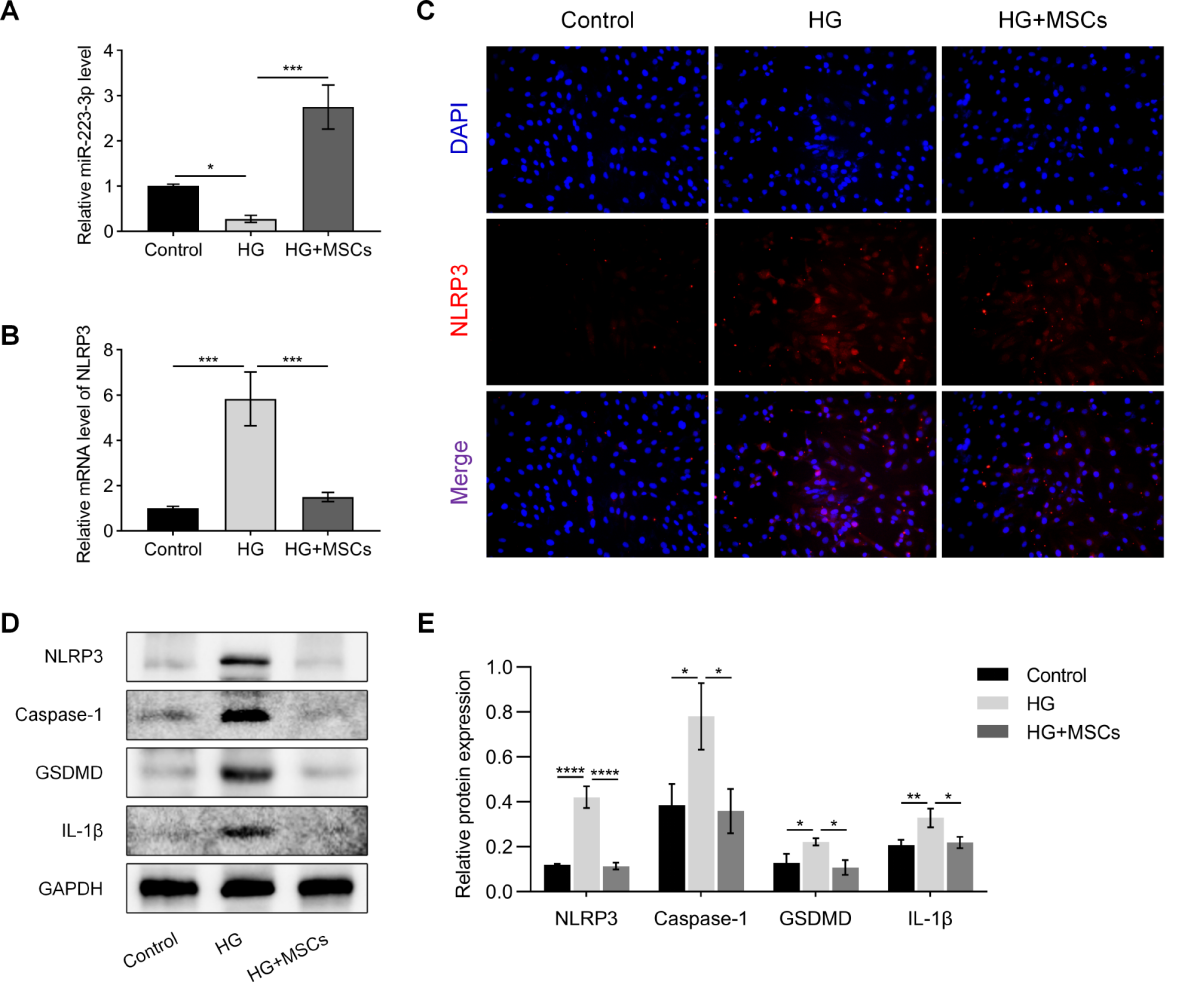
MSCs inhibit NLRP3-mediated pyroptosis in H9C2 cells treated with HG. (**A, B**) MSCs upregulate the expression of miR-223-3p and downregulate the expression of NLRP3 in H9C2 cells treated with HG. (**C**) Cell immunofluorescence staining showing that MSCs can reduce the protein expression of NLRP3 in H9C2 cells treated with HG. (**D, E**) Western blot assays showed that MSCs reduced the protein levels of NLRP3, Caspase-1, GSDMD, and IL-1β in H9C2 cells treated with HG. (Control group: normal glucose, 5 mmol/L; HG group: high glucose, 33 mmol/L; HG + MSCs group: high glucose + MSC treatment)

We utilized online bioinformatics analysis software (http://www.targetscan.org) and identified the presence of a binding site for miR-223-3p in the 3’-UTR of NLRP3 (Fig. 3A). Additionally, qRT-PCR detection in the myocardium revealed significant downregulation of miR-223-3p expression in mice with DCM, while miR-223-3p expression was substantially upregulated in the group treated with MSCs (Fig. 3B). Structural validation of the interaction between miR-223-3p and NLRP3 was performed using a dual-luciferase reporter gene assay. The results showed that in the NLRP3-WT (wild-type) group, the relative luciferase activity of H9C2 cells cotransfected with miR-223-3p was significantly inhibited, while no inhibition was observed in the NLRP3-MUT (mutant) group (Fig. 3C and E). Subsequently, functional validation of the miR-223-3p/NLRP3 interaction was conducted by transfecting mimics or inhibitors into H9C2 cells. Compared with transfection with the miRNA NC, transfection with the miR-223-3p mimic decreased the expression of NLRP3, whereas transfection with the miR-223-3p inhibitor increased the expression of NLRP3 (Fig. 3F). Compared to transfection with the miRNA NC, transfection with the miR-223-3p mimic caused a significant downregulation of NLRP3 expression and a reduction in the level of the NLRP3 protein. Conversely, transfection with a miR-223-3p inhibitor resulted in a notable upregulation of NLRP3 expression and an increase in the protein level of NLRP3 (Fig. 4G and H). The data presented robust evidence supporting the targeted binding regulation of miR-223-3p to NLRP3 in H9C2 cells.

### MSCs inhibit NLRP3-mediated pyroptosis in H9C2 cells treated with HG

In vitro experiments were conducted by subjecting H9C2 cells to HG treatment to simulate myocardial cell injury and coculturing them with MSCs for therapeutic purposes. The results demonstrated a significant decrease in the expression of miR-223-3p and a notable increase in NLRP3 expression in the experimental group treated with HG compared to the control group. However, upon coculture with MSCs, there was a substantial increase in miR-223-3p expression and a marked decrease in NLRP3 expression (Fig. 4A and B). Immunofluorescence staining revealed a significant increase in the fluorescence signal of the NLRP3 protein in the HG group compared to that in the control group. However, this signal was mitigated in the HG + MSCs group (Fig. 4C). Additionally, Western blot assays demonstrated substantial upregulation of the NLRP3 protein in the HG group, accompanied by notable increases in the levels of the Caspase-1, GSDMD, and IL-1β proteins. Conversely, in the HG + MSCs group, these protein levels were effectively decreased (Fig. 4D and E). These findings suggest that MSCs can suppress NLRP3-mediated pyroptosis in H9C2 cells treated with HG, and it is plausible that miR-223-3p plays a crucial regulatory role in this intricate process.

### MSCs stimulate the miR-223-3p/NLRP3 pathway to attenuate inflammation and pyroptosis

**Fig. 5:**
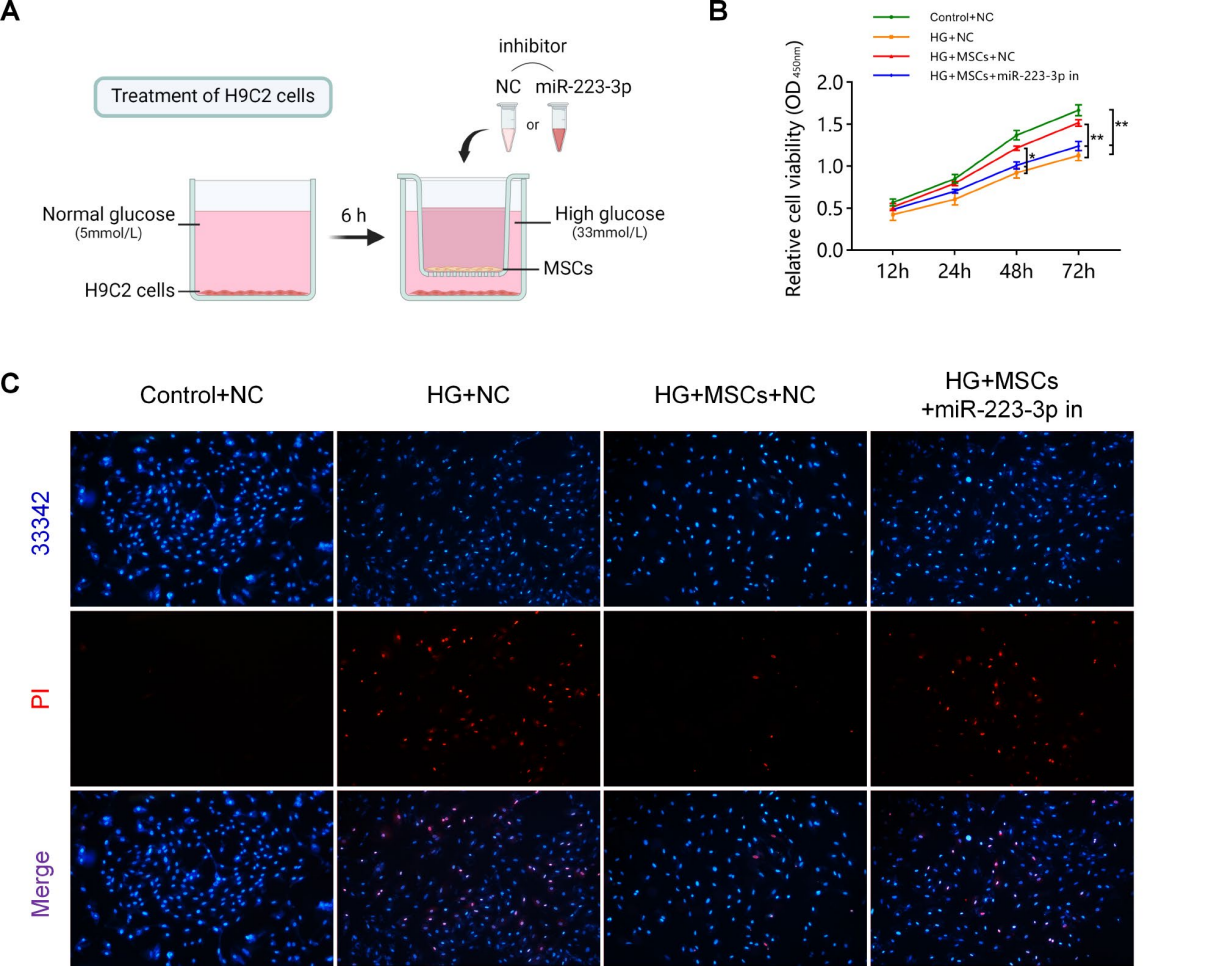
MSCs enhance cell viability and maintain cell survival in H9C2 cells treated with HG. (**A**) Schematic diagram of the treatment of H9C2 cells and MSCs in a coculture system. (**B**) The CCK-8 assay revealed that MSCs enhanced the survival rate of H9C2 cells under HG conditions. However, this beneficial effect was attenuated upon the introduction of a miR-223-3p inhibitor. (**C**) The miR-223-3p inhibitor impeded the ability of MSCs to sustain H9C2 cell survival under HG conditions. (Control + NC group: normal glucose + inhibitor NC; HG + NC group: high glucose + inhibitor NC; HG + MSCs + NC group: high glucose + MSC treatment + inhibitor NC; HG + MSCs + miR-223-3p inhibitor group: high glucose + MSC treatment + miR-223-3p inhibitor)

To investigate the regulatory effect of MSCs on the miR-223-3p/NLRP3 pathway, we conducted a coculture experiment involving MSCs and H9C2 cells transfected with either a miR-223-3p inhibitor or NC (Fig. 5A). First, we performed cell viability assays. The results revealed that cells in the Control + NC group exhibited robust cell viability, whereas cell viability was significantly suppressed in both the HG + NC group and HG + MSCs + miR-223-3p inhibitor group. However, an improvement in cell viability was observed in the HG + MSCs + NC group (Fig. 5B). As shown in Fig. 5C, the Control + NC group exhibited few PI-positive cells, whereas both the HG + NC and HG + MSCs + miR-223-3p inhibitor groups demonstrated a significant increase in PI-positive cells. In contrast, the HG + MSCs + NC group displayed a noteworthy reduction in the number of PI-positive cells.

**Fig. 6:**
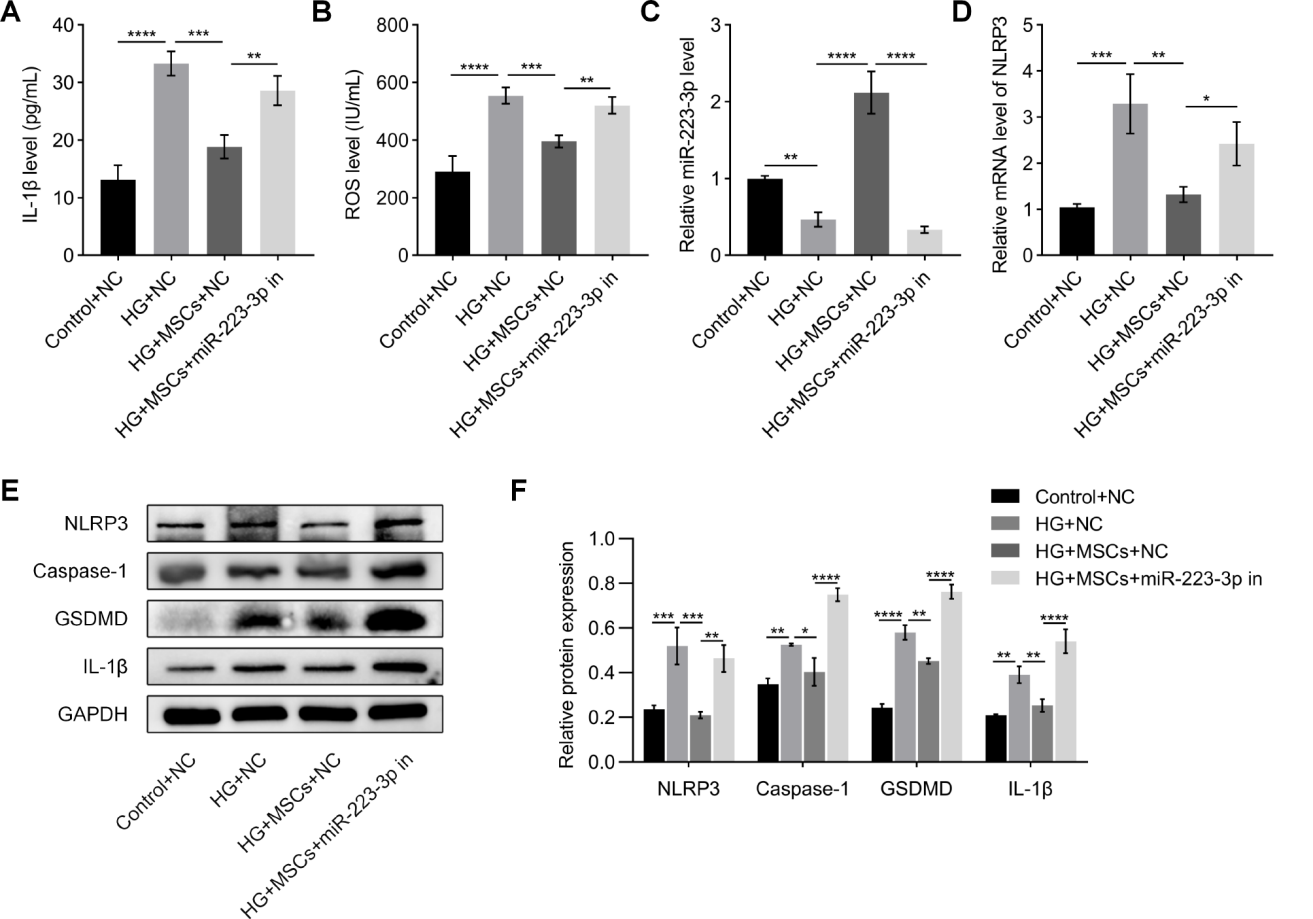
MSCs stimulate the miR-223-3p/NLRP3 pathway to attenuate inflammation and pyroptosis. (**A, B**) After transfection with the miR-223-3p inhibitor, MSCs failed to attenuate the release of inflammatory factors such as IL-1β and ROS in H9C2 cells treated with HG. (**C, D**) The miR-223-3p inhibitor counteracted the upregulation effect of MSCs on miR-223-3p in H9C2 cells under HG conditions, leading to an increase in NLRP3 expression levels and (**E, F**) promoting the elevation of NLRP3, Caspase-1, GSDMD, and IL-1β protein levels

Furthermore, the ELISA results indicated a significant increase in the levels of IL-1β and ROS in both the HG + NC group and the HG + MSCs + miR-223-3p inhibitor group. Conversely, a decreasing trend was observed for these indicators in the HG + MSCs + NC group (Fig. 6A and B). The qRT-PCR results demonstrated a significant decrease in the expression of miR-223-3p and an increase in the expression of NLRP3 in both the HG + NC group and the HG + MSCs + miR-223-3p inhibitor group. However, this effect was reversed in the HG + MSCs + NC group (Fig. 6C and D). The results of the Western blot assays demonstrated that, in comparison to those in the Control + NC group, both the HG + NC group and the HG + MSCs + miR-223-3p inhibitor group exhibited upregulated levels of the NLRP3, Caspase-1, GSDMD, and IL-1β proteins. However, a decreasing trend in the expression of these proteins was observed in the HG + MSCs + NC group compared to the other two groups (Fig. 6E and F). The above data demonstrate that MSCs effectively ameliorate the inflammatory response and pyroptosis in HG-treated H9C2 cells by upregulating miR-223-3p and downregulating NLRP3. However, this beneficial effect is reversed upon transfection with a miR-223-3p inhibitor.

## Discussion

DCM, a cardiovascular disease associated with diabetes, is characterized by cardiac insufficiency in diabetic patients without the presence of other comorbidities such as coronary artery disease (CAD), uncontrolled hypertension, severe valvular heart disease, or congenital heart disease (CHD). This pathological process was initially described by Rubler et al. and remained an important area of research in the field of cardiology [32, 33]. According to statistical data, the global prevalence of diabetes was approximately 500 million individuals in 2019, and it is projected to increase by 25% by 2030 and by 51% by 2045 [34]. Currently, conventional therapies involve regulating blood glucose levels and slowing the deterioration of myocardial function by administering various medications, including angiotensin-converting enzyme inhibitors (ACEIs), angiotensin receptor blockers (ARBs), beta-blockers (BBs), and aldosterone antagonists (AAs). However, these therapeutic approaches fail to mitigate the pathological changes occurring within the myocardium itself [3, 4]. Hence, it is crucial to explore innovative therapeutic strategies for dilated cardiomyopathy (DCM) that specifically focus on myocardial tissue.

**Fig. 7:**
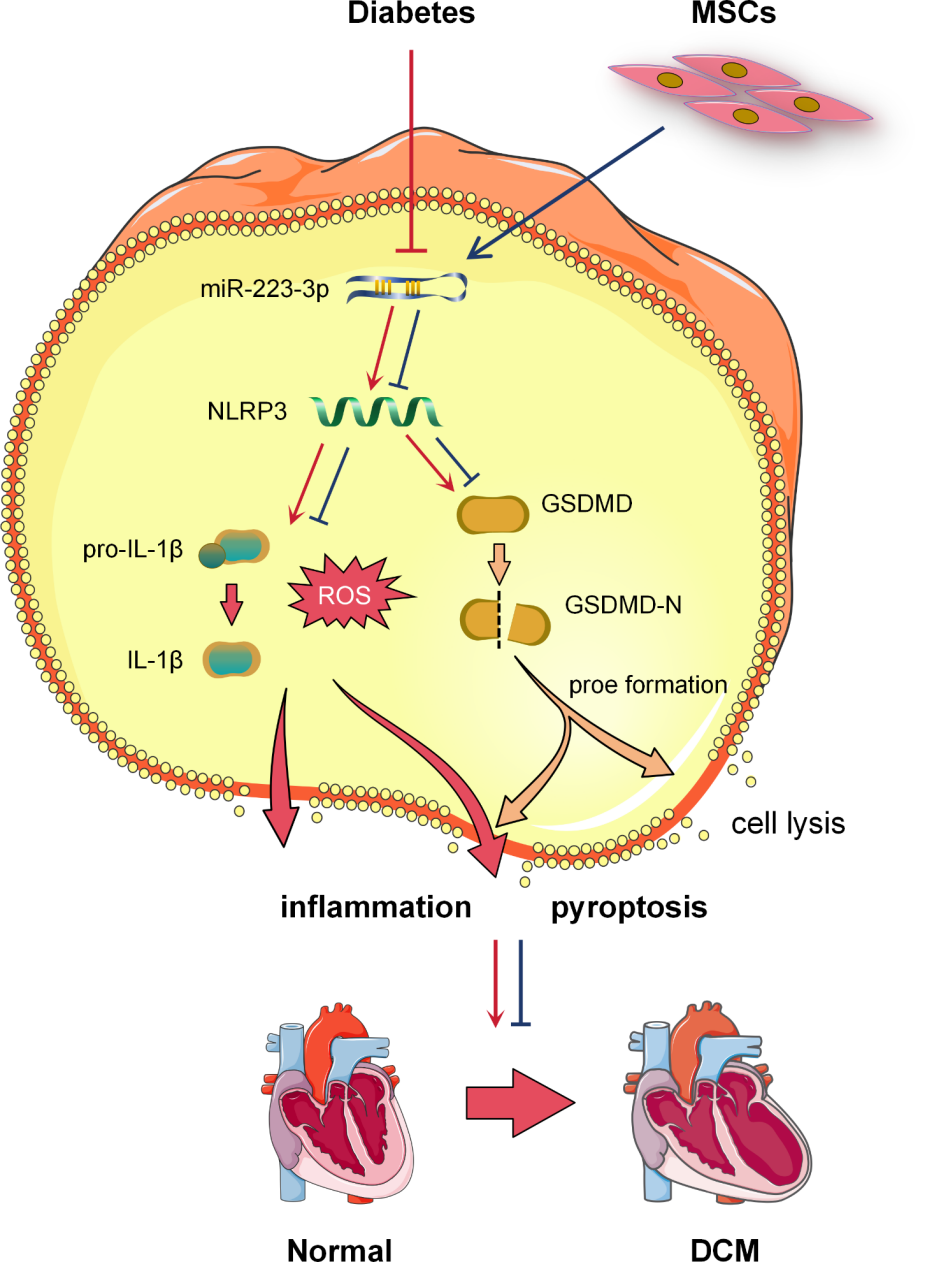
Mechanistic diagram elucidating the therapeutic potential of MSCs in DCM via modulation of the miR-223-3p/NLRP3 pathway. By modulating the regulatory impact of miR-223-3p on the NLRP3 gene, MSCs can effectively hinder abnormal activation of the NLRP3 pathway, diminish the production of associated proinflammatory factors (such as IL-1β and IL-18), and reduce GSDMD-mediated cell lysis. This ultimately protects myocardial cells and prevents the progression of DCM

MSC therapy holds tremendous potential as an advanced therapeutic approach for the management of DCM, demonstrating promising prospects across a diverse range of applications [9]. Extensive clinical and animal experimentation has consistently shown the capacity of MSCs to augment cardiac function in individuals suffering from DCM through multifaceted mechanisms [7, 8]. First, MSCs possess a remarkable capacity for self-renewal and differentiation, engrafting into the body and differentiating into pertinent tissues such as cardiomyocytes and vascular endothelial cells to actively participate in the repair of damaged areas [13]. Moreover, they can secrete a diverse array of growth factors and signaling molecules that effectively promote regeneration and restoration of surrounding tissues [11]. Second, during transplantation, MSCs also play a pivotal role in immune regulation to prevent detrimental consequences arising from hyperactive autoimmune reactions [12]. However, despite the immense potential demonstrated by MSC therapy, a comprehensive understanding of its precise theoretical mechanism remains elusive. To shed light on this matter, it is imperative to conduct further extensive investigations encompassing fundamental scientific research and clinical trials aimed at unraveling intricate details concerning the involved molecules, signaling pathways, and interaction networks. This study demonstrated that treatment with MSCs significantly enhances the expression of miR-223-3p in myocardial cells, resulting in the suppression of NLRP3 gene expression. As a consequence, this effectively reduces the production of various inflammatory factors such as IL-1β and ROS, as well as GSDMD protein-mediated cell lysis (Fig. 7). These findings unveil the pivotal role played by MSCs in regulating inflammation and pyroptosis processes in myocardial cells, thereby providing a novel theoretical foundation for MSC therapy in DCM.

Initially, we evaluated the efficacy of MSCs in the treatment of DCM using a mouse model in vivo. Diabetes was induced in mice by a high-glucose and high-fat diet combined with STZ injection. A blood glucose concentration ≥ 16.6 mM was considered to indicate successful induction. Starting from the 16th week, a regimen of MSC administration will be implemented via tail vein injection for treatment every two weeks, resulting in a total of three consecutive treatments. At the 24th week, an evaluation will be conducted to assess cardiac function and the extent of myocardial injury in all mice. On echocardiography, the LVIDD and LVIDS serve as indicators of changes in the left ventricular structure, while the LVEF and FS provide insight into the contraction function of the left ventricle. The E’/A’ ratio is a valuable measure of diastolic function within the left ventricle. These parameters are widely utilized and essential for accurate diagnosis, effective treatment, and comprehensive follow-up care. As shown in Fig. 1, an enlarged left ventricular structure and impaired systolic and diastolic function were observed in DCM model mice. After treatment with MSCs, these aberrations were effectively ameliorated and improved.CK and CK-MB levels are also commonly used as indicators to evaluate the degree of myocardial injury clinically. After MSC treatment, the levels of both indicators were significantly reduced. This therapeutic effect has also been observed in histological analysis of myocardial tissue. These findings substantiate the substantial efficacy of MSCs in the treatment of DCM; nevertheless, further investigation is imperative to elucidate their precise mechanism of action.

Recent findings have suggested a close association between the progression of DCM and the levels of inflammation and pyroptosis mediated by NLRP3, which has emerged as a highly focused area of investigation [35, 36]. NLRP3 is a crucial initiator of pyroptosis, as its activation can facilitate the maturation of Caspase-1, GSDMD, and IL-1β. This leads to the formation of cell membrane pores, resulting in cell lysis and death, as well as the release of inflammatory factors that trigger a cascade effect. However, there is currently no available literature on the potential regulatory role of MSC therapy in this process. To validate this hypothesis, we evaluated the inflammatory response and pyroptosis in DCM mice treated with or without MSCs. The results demonstrated a significant upregulation of NLRP3 in DCM mice, which was attenuated following MSC treatment. Additionally, MSCs reduced the release of inflammatory factors such as IL-1β and ROS and decreased the expression levels of pyroptosis-related proteins, including NLRP3, Caspase-1, and GSDMD (Fig. 2). The provided data demonstrate the efficacy of MSCs in attenuating inflammation and pyroptosis in mice with DCM through the modulation of NLRP3.

To further investigate the molecular mechanism by which MSCs inhibit NLRP3 expression, we reviewed several studies and found that MSCs play an important role in regulating host-derived microRNAs, which can in turn regulate gene expression in host cells [27, 28]. Through bioinformatics prediction analysis, we identified a targeted binding relationship between miR-223-3p and NLRP3. This discovery was substantiated through rigorous dual-luciferase reporter assays and gene transfection experiments (Fig. 3). Additionally, the expression of miR-233-3p decreased in DCM mice, but was subsequently restored upon treatment with MSCs (Fig. 3B). As shown in Fig. 3C, miR-223-3p has a clear binding site with wild-type NLRP3. In the dual-luciferase assay, miR-223-3p significantly inhibited the fluorescence signal of wild-type NLRP3, but it could not inhibit the fluorescence signal of mutant NLRP3. Subsequently, we used miR-223-3p mimics and inhibitors to verify their interaction. As shown in Fig. 3F-H, the miR-223-3p mimics and inhibitors effectively regulated the gene expression and protein levels of NLRP3 in H9C2 cells. However, this interaction’s regulation of downstream molecules and cellular functions requires further validation.

Studies have shown that high glucose culture conditions significantly induce pyroptosis in myocardial cells by increasing inflammatory factors, reactive oxygen species, and cell membrane damage [11, 23]. This process involves the activation of inflammasomes (such as the NLRP3 inflammasome) and the formation of cell membrane pores mediated by Caspase-1, ultimately leading to reduced cell viability and even cell death [19, 20]. In this study, in vitro experiments found that MSCs significantly inhibited high glucose-induced NLRP3 gene expression and protein levels in H9C2 cardiomyocytes (Fig. 4B and C). Additionally, qPCR revealed that miR-223-3p expression decreased under high glucose conditions, while MSCs enhanced its expression. This result is consistent with in vivo experiments. Subsequently, we further examined the levels of downstream pyroptosis-related proteins mediated by NLRP3/Caspase-1. As shown in Fig. 4D and E, MSCs significantly reduced the protein levels of Caspase-1, GSDMD, and IL-1β induced by high glucose. Furthermore, we assessed the viability and survival of cardiomyocytes under different treatments (Fig. 5B and C). Under high glucose conditions, the viability of H9C2 cells significantly decreased, and more cells died. However, this situation improved when co-cultured with MSCs, as MSCs mitigated this effect by reducing cell pyroptosis. The counteraction of this improvement by the miR-223-3p inhibitor once again underscored the critical role of miR-223-3p in regulating cell pyroptosis. These results indicate that MSCs can effectively prevent myocardial cell pyroptosis under high glucose conditions through the miR-223-3p/NLRP3 pathway, thereby exerting a protective effect.

Pyroptosis is a form of inflammation-mediated programmed cell death [17, 18]. Under high glucose conditions, the level of ROS in cardiomyocytes increases, leading to oxidative stress and inducing the expression and release of inflammatory factors such as IL-1β [20, 21]. These factors significantly induce the activation of the NLRP3 inflammasome and the formation of cell membrane pores mediated by Caspase-1/GSDMD in H9C2 cells, ultimately resulting in the release of cell contents and cell death. As shown in Fig. 6A and B, IL-1β and ROS levels were significantly elevated in the HG + NC group. However, MSCs enhanced the expression of miR-223-3p in H9C2 cells, which targeted and inhibited the expression of NLRP3 (Fig. 6C and D), thereby suppressing the pyroptosis process, reducing the release of inflammatory factors, and lowering ROS levels.

It is well known that the interaction between miRNA and target genes is achieved through the RNA interference mechanism [24, 25]. In this study, miR-223-3p was found to complementarily pair with the 3’ untranslated region of the NLRP3 gene, thereby mediating its silencing and reducing the synthesis of NLRP3 protein. This action can regulate the formation and activation of inflammasomes. NLRP3 inflammasome is an intracellular multiprotein complex composed mainly of NLRP3, ASC, and Caspase-1, which are involved in initiating and regulating inflammatory responses, leading to the maturation and release of pro-inflammatory cytokines such as IL-1β and IL-18 [29, 30]. Caspase-1 is a key component of the NLRP3 inflammasome. During the activation of the NLRP3 inflammasome, Caspase-1 is activated to cleave and activate GSDMD, releasing its N-terminal fragment. The N-terminal fragment of GSDMD forms cell membrane pores, leading to pyroptosis and the release of pro-inflammatory mediators (such as IL-1β and IL-18), thereby exacerbating the inflammatory response. The action of miR-223-3p reduces the formation of NLRP3 inflammasomes, thereby inhibiting pyroptosis and excessive inflammatory responses.

Subsequently, we conducted a rescue experiment using a miR-223-3p inhibitor to suppress the expression of miR-223-3p in cardiomyocytes. The results showed that the miR-223-3p inhibitor negated the therapeutic effects of MSCs, failing to inhibit pyroptosis and the release of intracellular inflammatory factors, and exacerbating oxidative stress. This phenomenon was also confirmed by Western blot analysis of downstream Caspase-1, GSDMD, and IL-1β (Fig. 6E and F). These results indicate that MSCs reduce the release of inflammatory factors and lower ROS levels by ameliorating pyroptosis in H9C2 cells, thereby providing a favorable environment for cell survival.

However, despite the valuable insights and methodologies offered by this discovery for the management of DCM, certain limitations necessitate attention. In high-glucose-induced myocardial cell damage, MSCs exhibit significant anti-pyroptotic capabilities, achieved through regulation of the miR-223-3p/NLRP3 pathway. This regulatory mechanism is complex and may involve pathways such as the release of miRNA via exosomes, modulation of inflammatory responses, and intercellular transcriptional regulation [26, 37]. Studies have shown that MSCs release exosomes or extracellular vesicles containing miRNA, which participate in regulating the transcription and translation of target genes in recipient cells [8, 38]. MSCs themselves possess anti-inflammatory properties, influencing the expression of miRNA and mRNA in target cells by secreting anti-inflammatory factors or modulating the release of inflammatory mediators [6, 7]. Furthermore, MSCs may affect gene transcription and translation in target cells through released cytokines or direct cell-cell contact [27].

In this study, we found that miR-223-3p, as a regulator of the pyroptosis molecule NLRP3, is downregulated in the myocardium of diabetic cardiomyopathy mice, but significantly increased after MSC treatment, thereby reducing the protein levels of NLRP3, Caspase-1, GSDMD, and IL-1β. All data in this study are derived from original research and are reliable. This contributes significantly to enriching the theoretical basis for MSC therapy in myocardial cell injury. Unfortunately, due to experimental constraints and time limitations, we were unable to fully elucidate the detailed pathways through which MSCs regulate miR-223-3p in myocardial cells. However, this also points us toward future research directions, providing hope for the theoretical support of developing new stem cell therapy strategies.

## Conclusion

In summary, the results of this study demonstrate that MSCs play a crucial role in ameliorating inflammation and pyroptosis in myocardial cells. By mediating the regulatory effect of miR-223-3p on the NLRP3 gene, MSCs can effectively inhibit aberrant activation of the NLRP3 pathway, reduce the production of related proinflammatory factors (such as IL-1β and ROS), and decrease GSDMD-mediated cell lysis. Consequently, the local inflammatory environment in cardiac tissue is alleviated, which helps protect damaged or at-risk myocardial cells. In conclusion, these findings reveal the significant potential of MSCs in treating heart-related issues and provide a theoretical basis for corresponding clinical practices. Further exploration of their mechanisms and optimization of therapeutic effects will contribute to achieving more remarkable outcomes in this field.

### Electronic supplementary material

Below is the link to the electronic supplementary material.

Supplementary Material 1

Supplementary Material 2

Supplementary Material 3

Supplementary Material 4

Supplementary Material 5

Supplementary Material 6

Supplementary Material 7

Supplementary Material 8

Supplementary Material 9

Supplementary Material 10

Supplementary Material 11

Supplementary Material 12

Supplementary Material 13

Supplementary Material 14

Supplementary Material 15

## Data Availability

No datasets were generated or analysed during the current study.

## Abbreviations

DCM: Diabetic Cardiomyopathy
MSCs: Mesenchymal Stem Cells
NLRP3: Nucleotide-Binding Oligomerization Domain, Leucine-Rich Repeat, and Pyrin Domain-Containing Protein 3
GSDMD: Gasdermin D
IL-1β: Interleukin-1β
ROS: Reactive Oxygen Species
miRNA: Micro RNA
STZ: Streptozotocin
LVIDD: Left Ventricular End-Diastolic Internal Diameter
LVIDS: Left Ventricular End-Systolic Internal Diameters
LVEF: Left Ventricular Ejection Fraction
FS: Fractional Shortening
E’/A’: The Early to Late Diastolic Mitral Annular Velocity
HW/BW: Heart Weight to Body Weight Ratio
CK/CK-MB: Creatine Kinase/ Creatine Kinase-MB
Col-I/Col-III: Collage-I/Collagen-III
HG: High Glucose
NC: Negative Control
