# Supplementary figures and images for: Mesenchymal stem cells ameliorate inflammation and pyroptosis in diabetic cardiomyopathy via the miRNA-223-3p/NLRP3 pathway
Source: Diabetol Metab Syndr. 2024 Jul 2;16:146. doi: 10.1186/s13098-024-01389-7 (PMC11221100; doi:10.1186/s13098-024-01389-7)

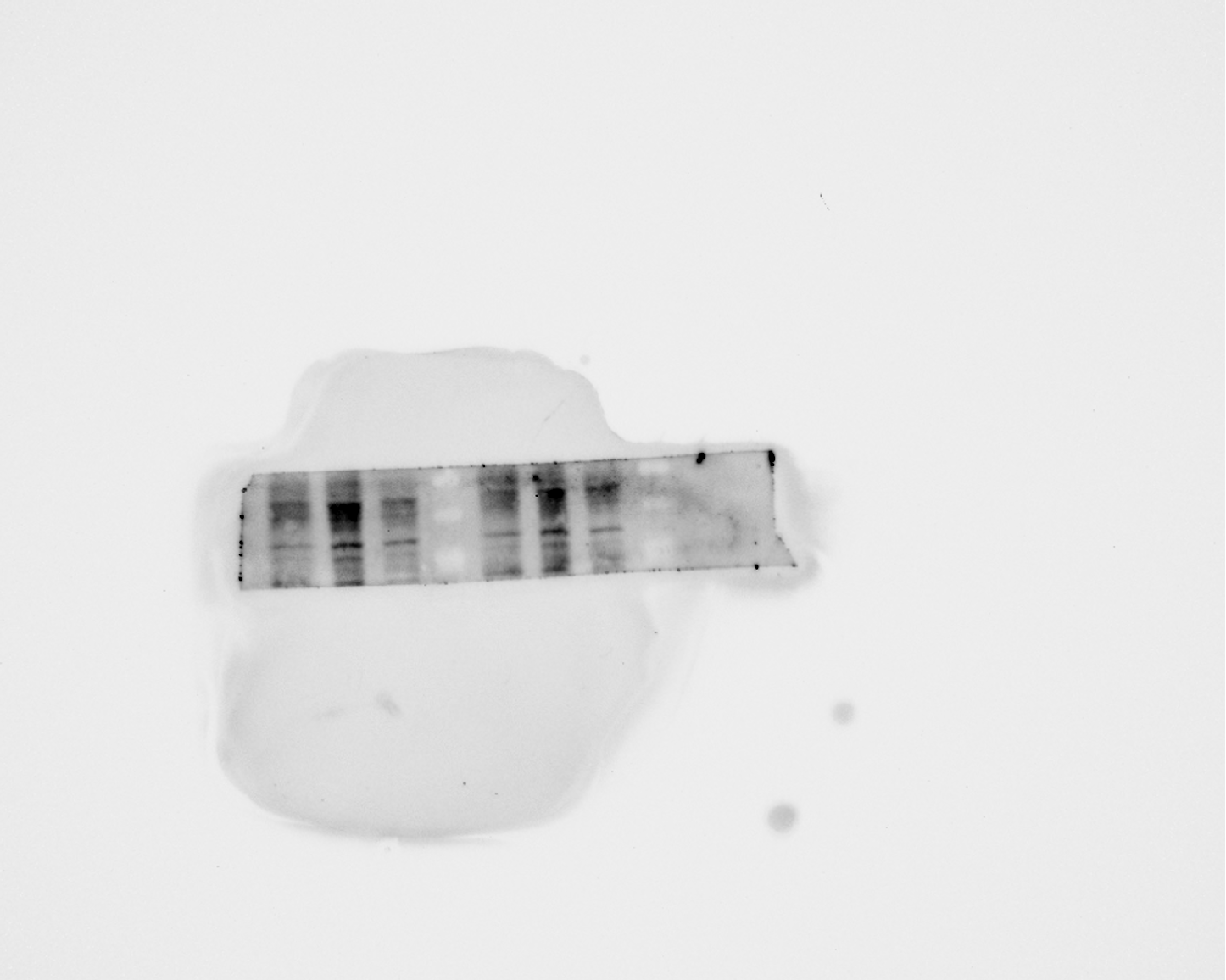

Supplement: Supplementary file 1 — Supplementary Material 1 [file 13098_2024_1389_MOESM1_ESM.tif]

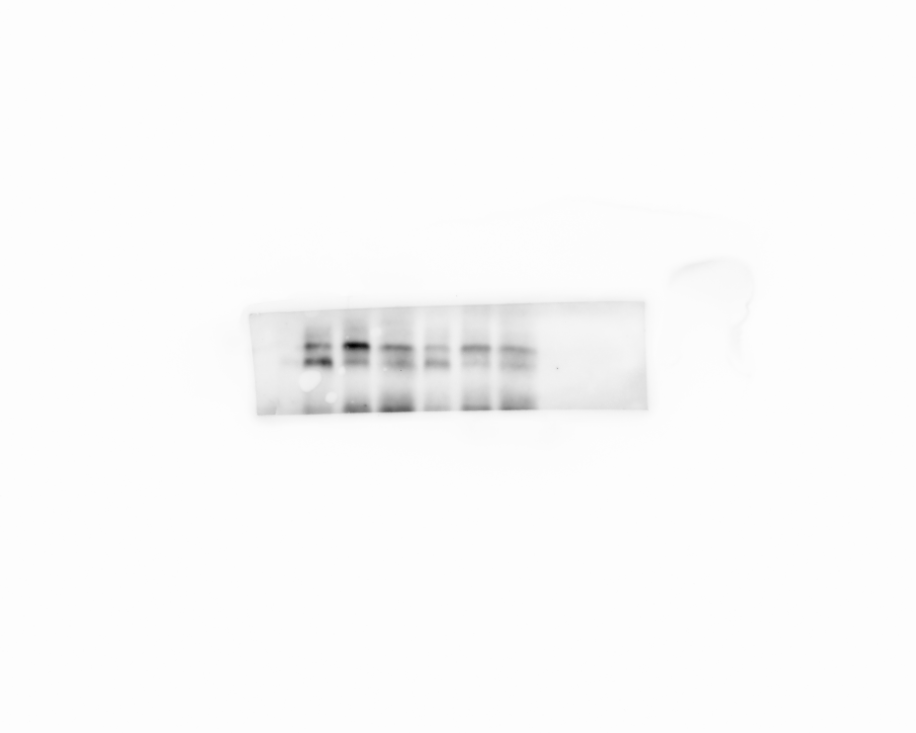

Supplement: Supplementary file 2 — Supplementary Material 2 [file 13098_2024_1389_MOESM2_ESM.tif]

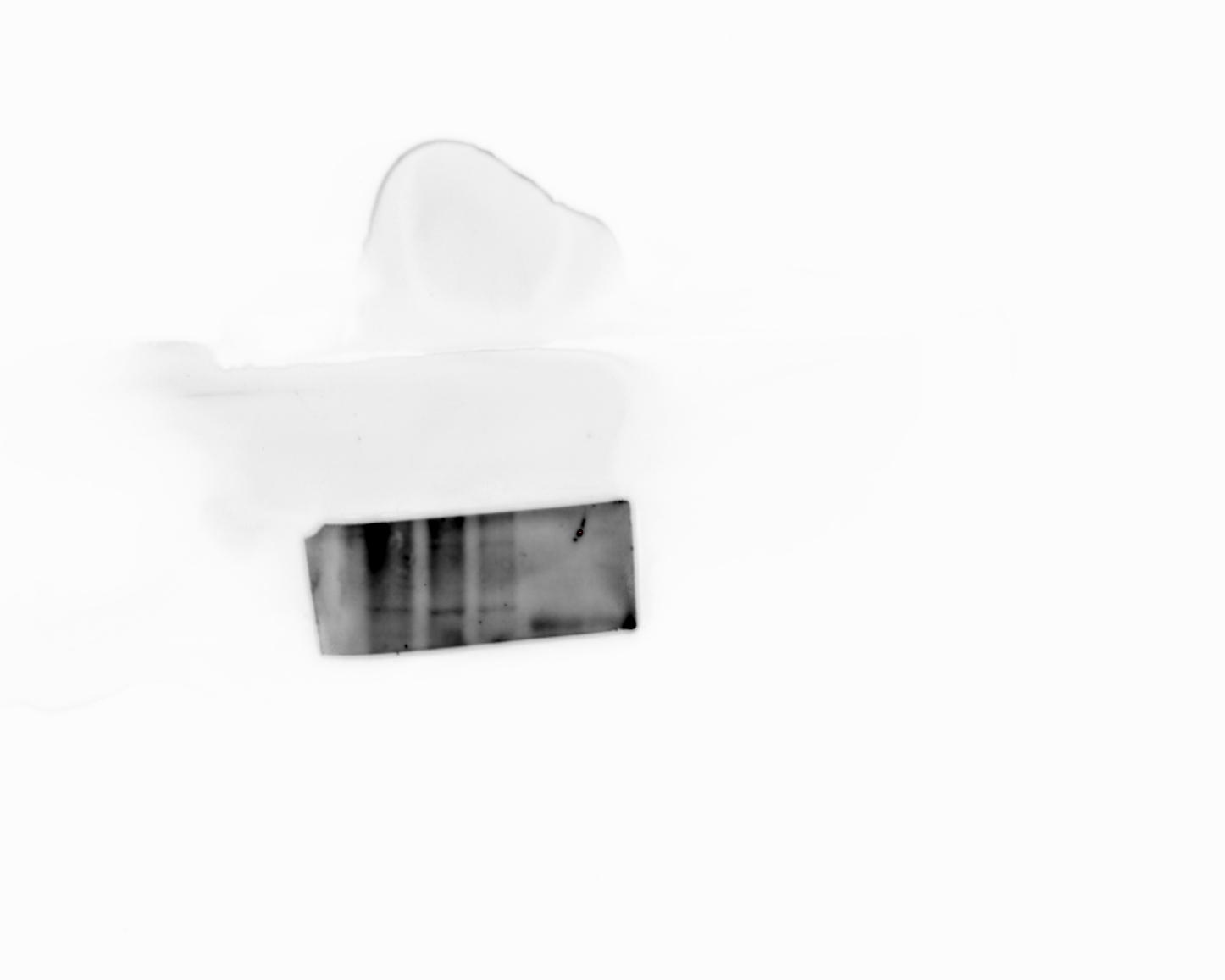

Supplement: Supplementary file 3 — Supplementary Material 3 [file 13098_2024_1389_MOESM3_ESM.tif]

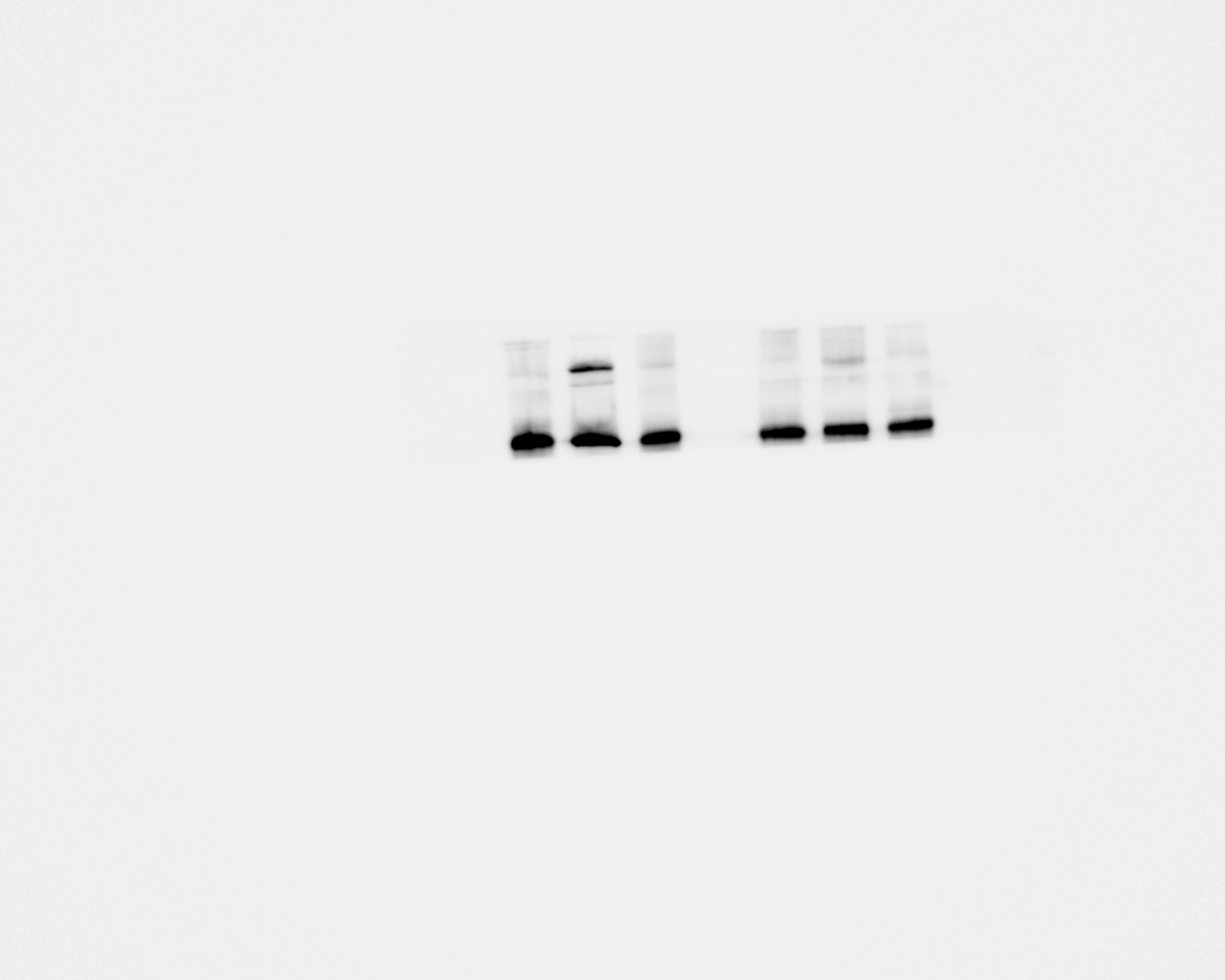

Supplement: Supplementary file 4 — Supplementary Material 4 [file 13098_2024_1389_MOESM4_ESM.tif]

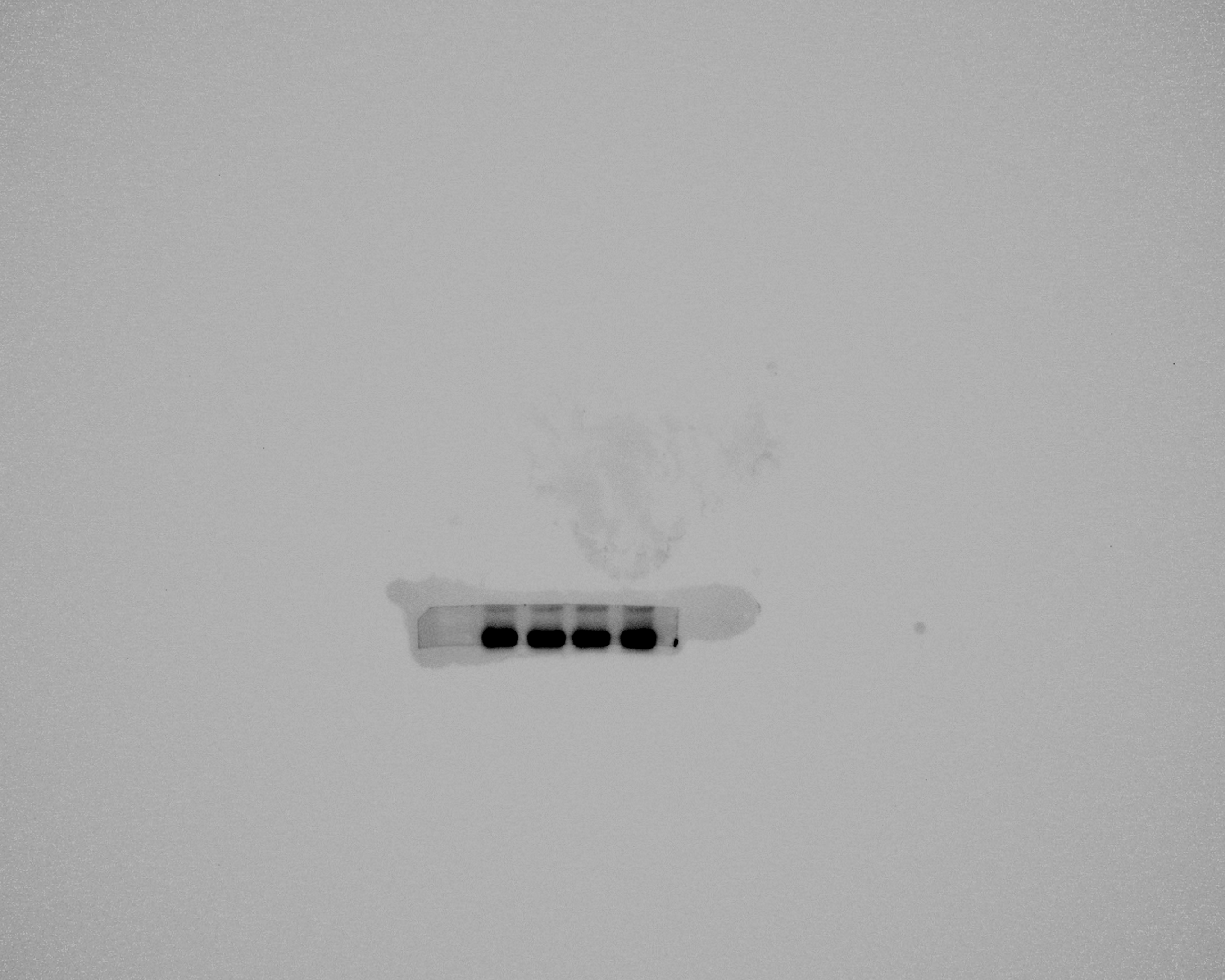

Supplement: Supplementary file 5 — Supplementary Material 5 [file 13098_2024_1389_MOESM5_ESM.tif]

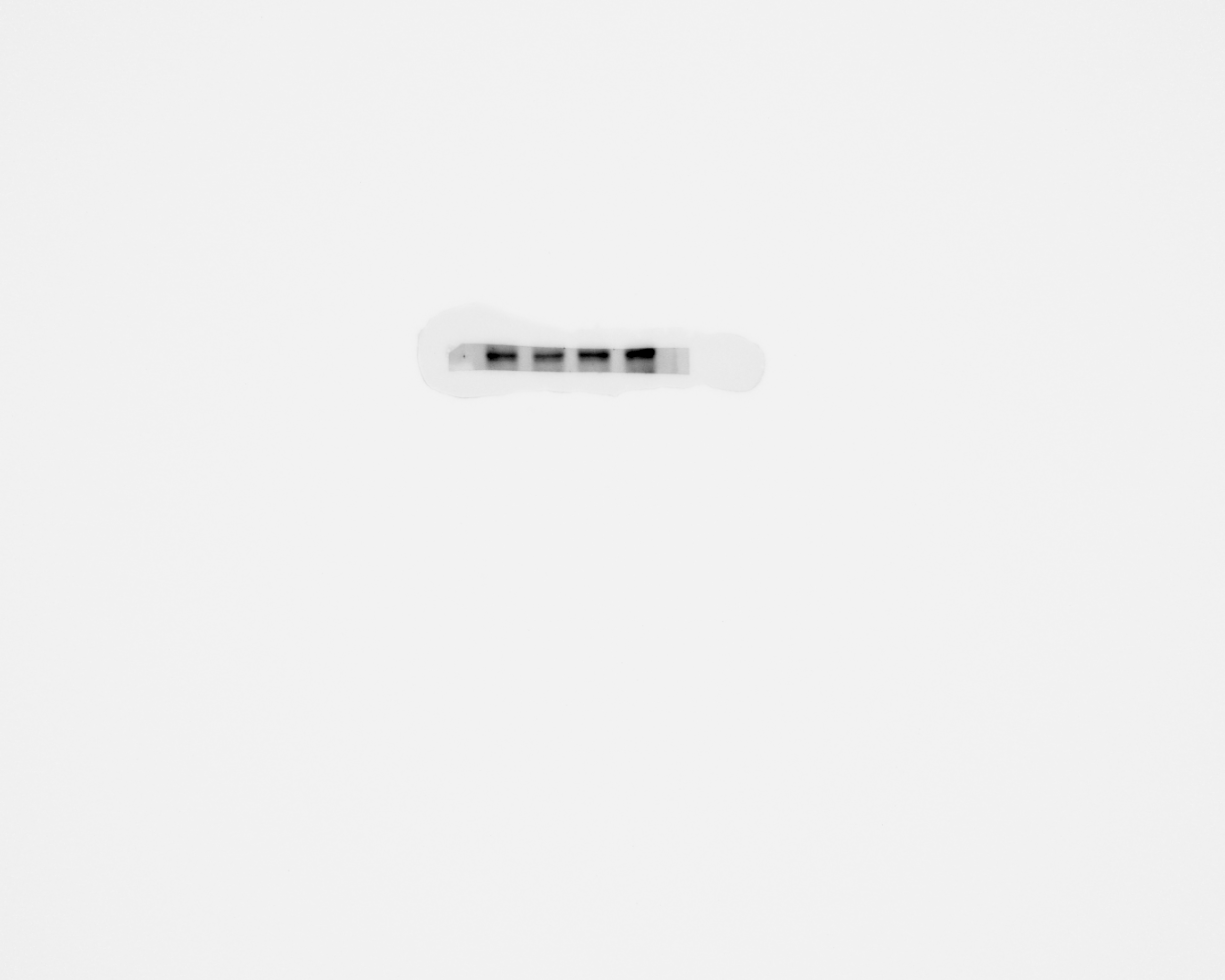

Supplement: Supplementary file 6 — Supplementary Material 6 [file 13098_2024_1389_MOESM6_ESM.tif]

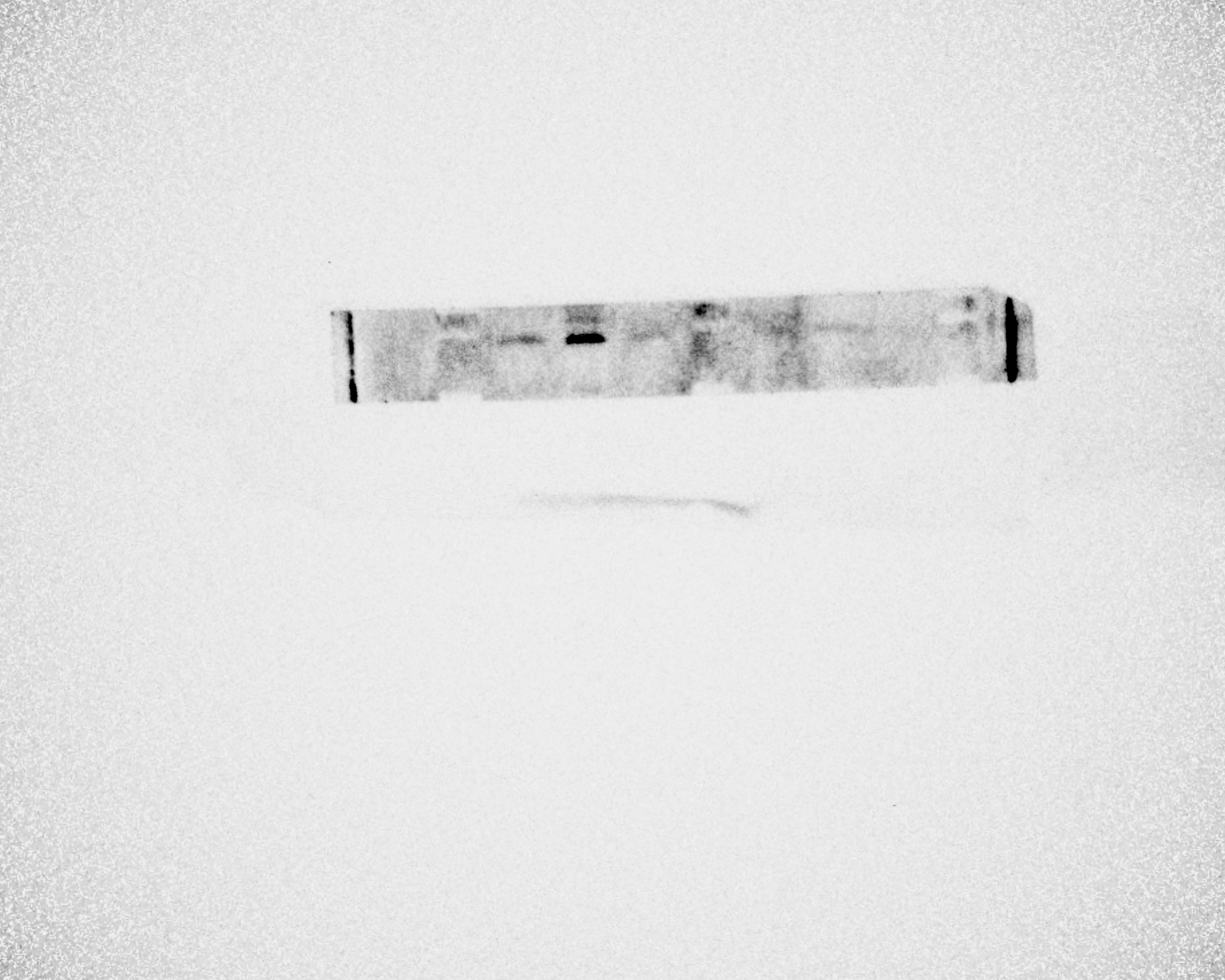

Supplement: Supplementary file 7 — Supplementary Material 7 [file 13098_2024_1389_MOESM7_ESM.tif]

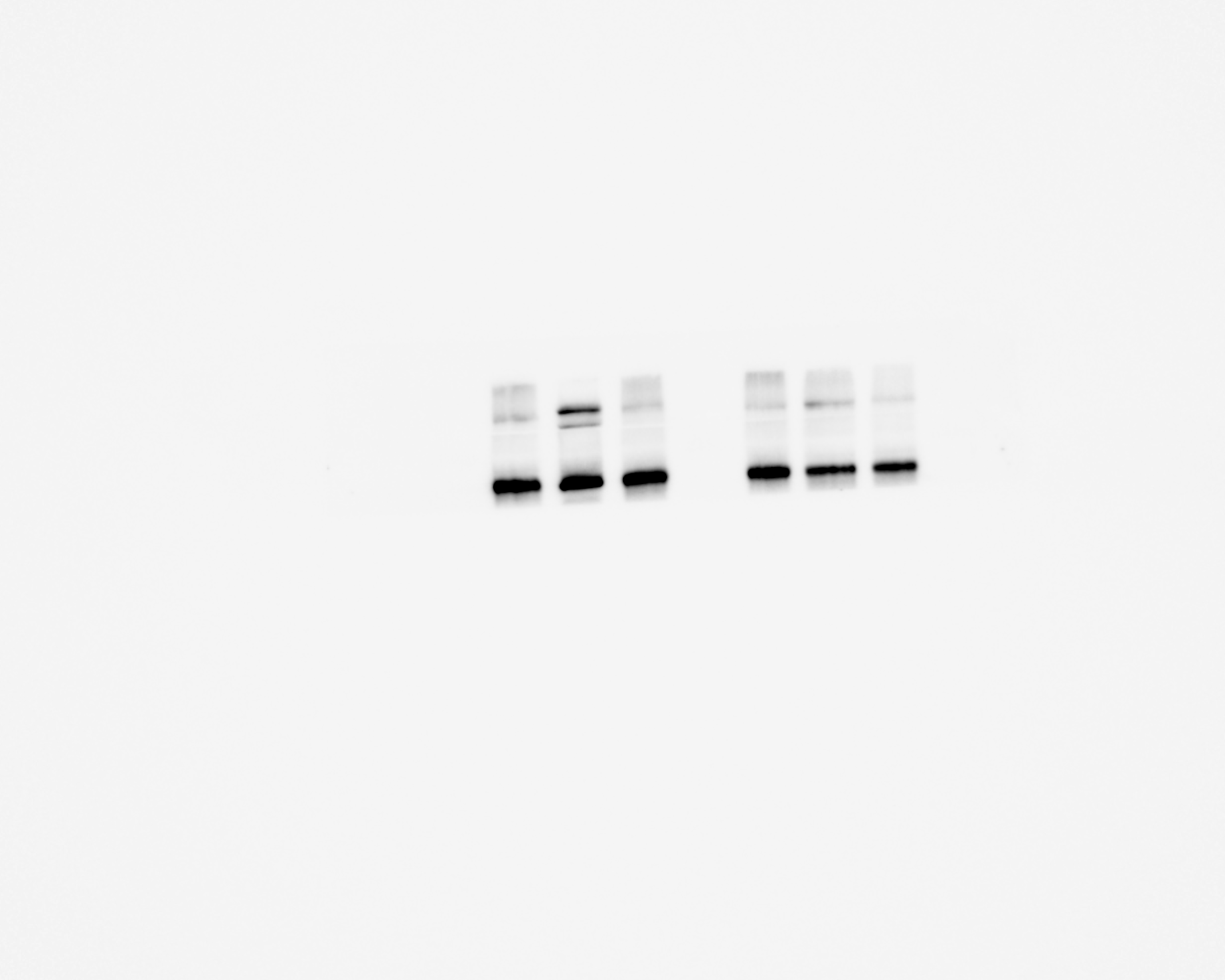

Supplement: Supplementary file 8 — Supplementary Material 8 [file 13098_2024_1389_MOESM8_ESM.tif]

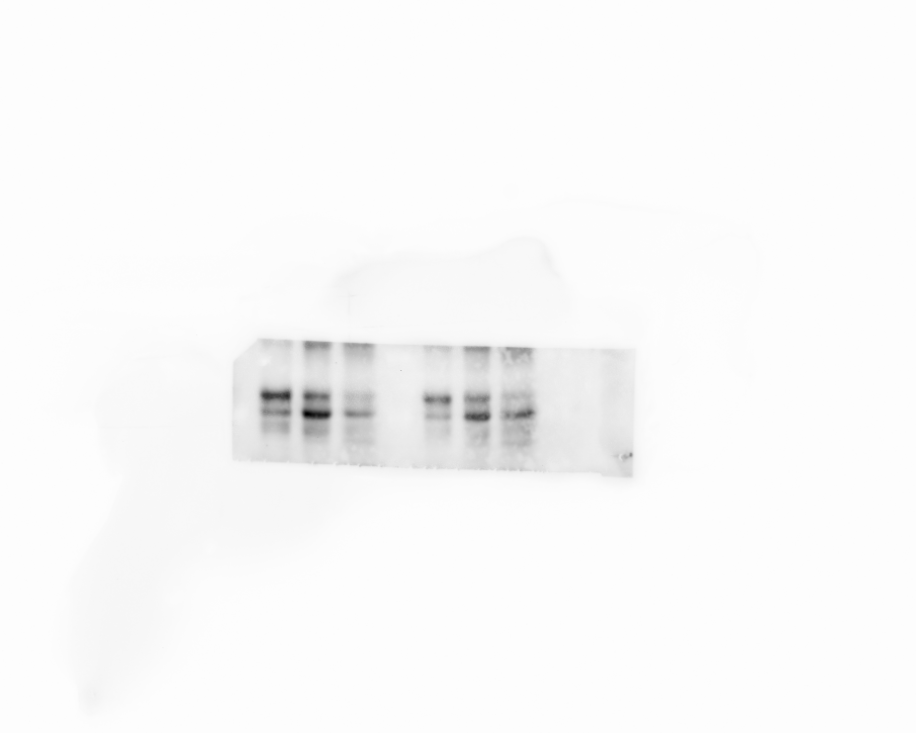

Supplement: Supplementary file 9 — Supplementary Material 9 [file 13098_2024_1389_MOESM9_ESM.tif]

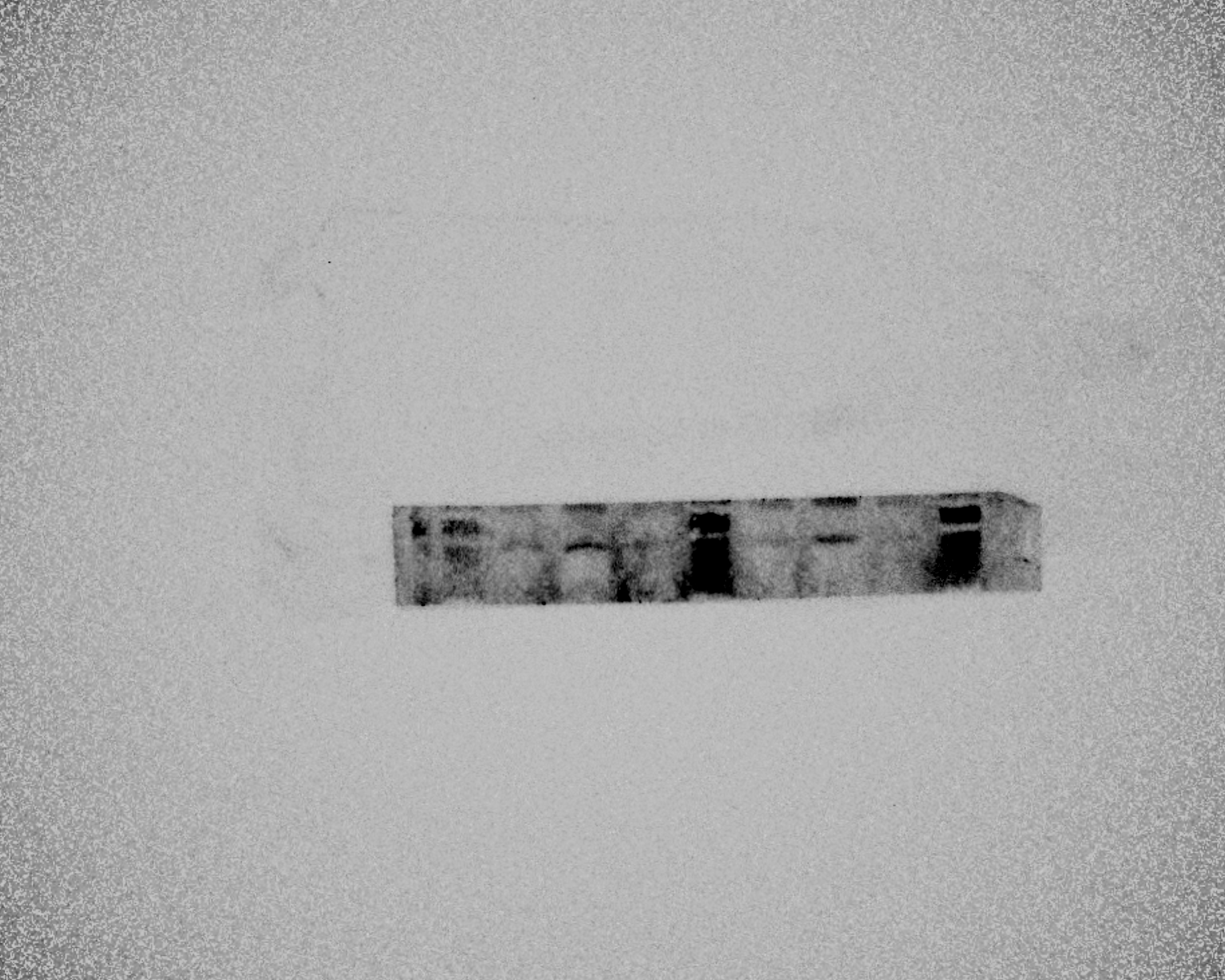

Supplement: Supplementary file 10 — Supplementary Material 10 [file 13098_2024_1389_MOESM10_ESM.tif]

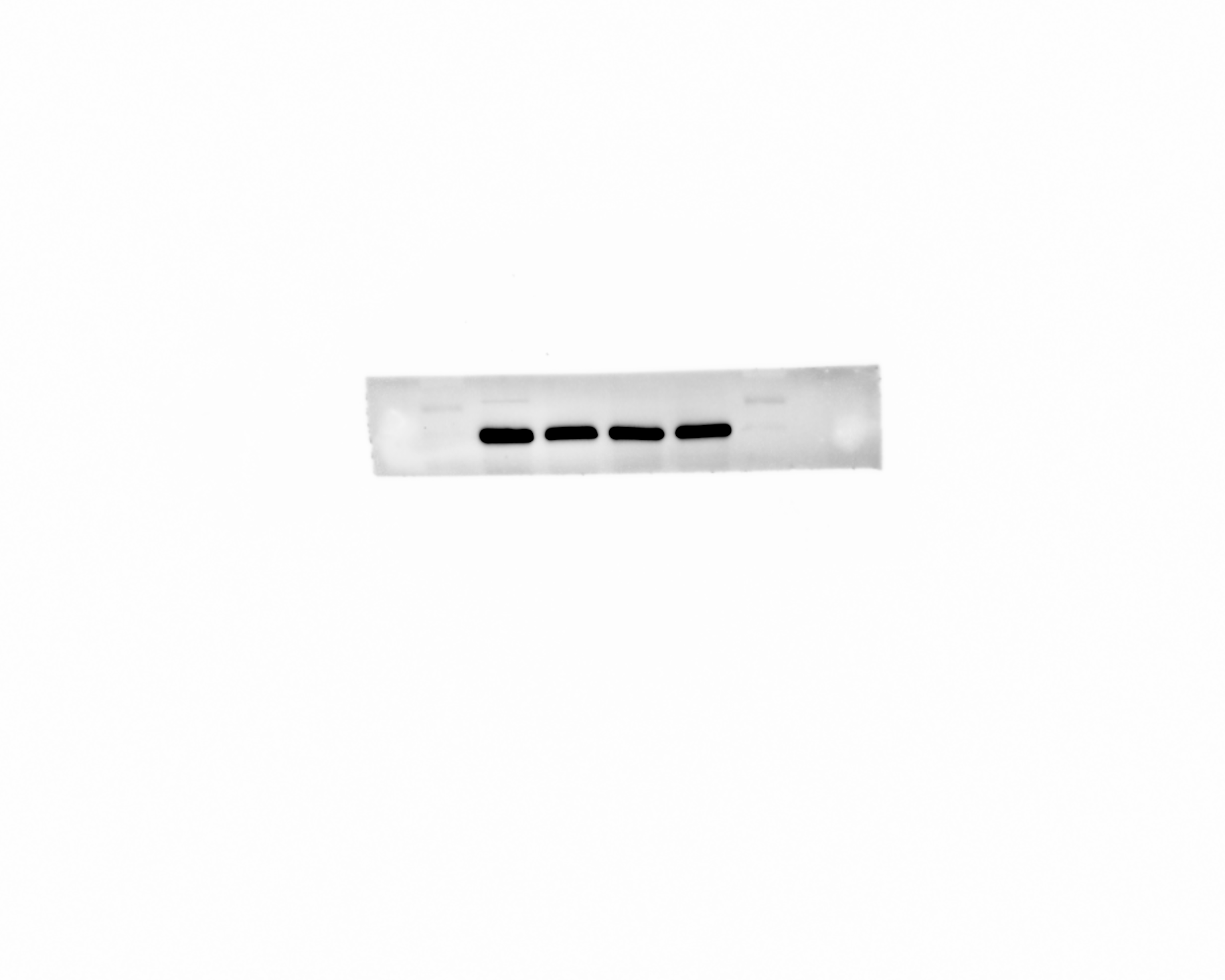

Supplement: Supplementary file 12 — Supplementary Material 12 [file 13098_2024_1389_MOESM12_ESM.tif]

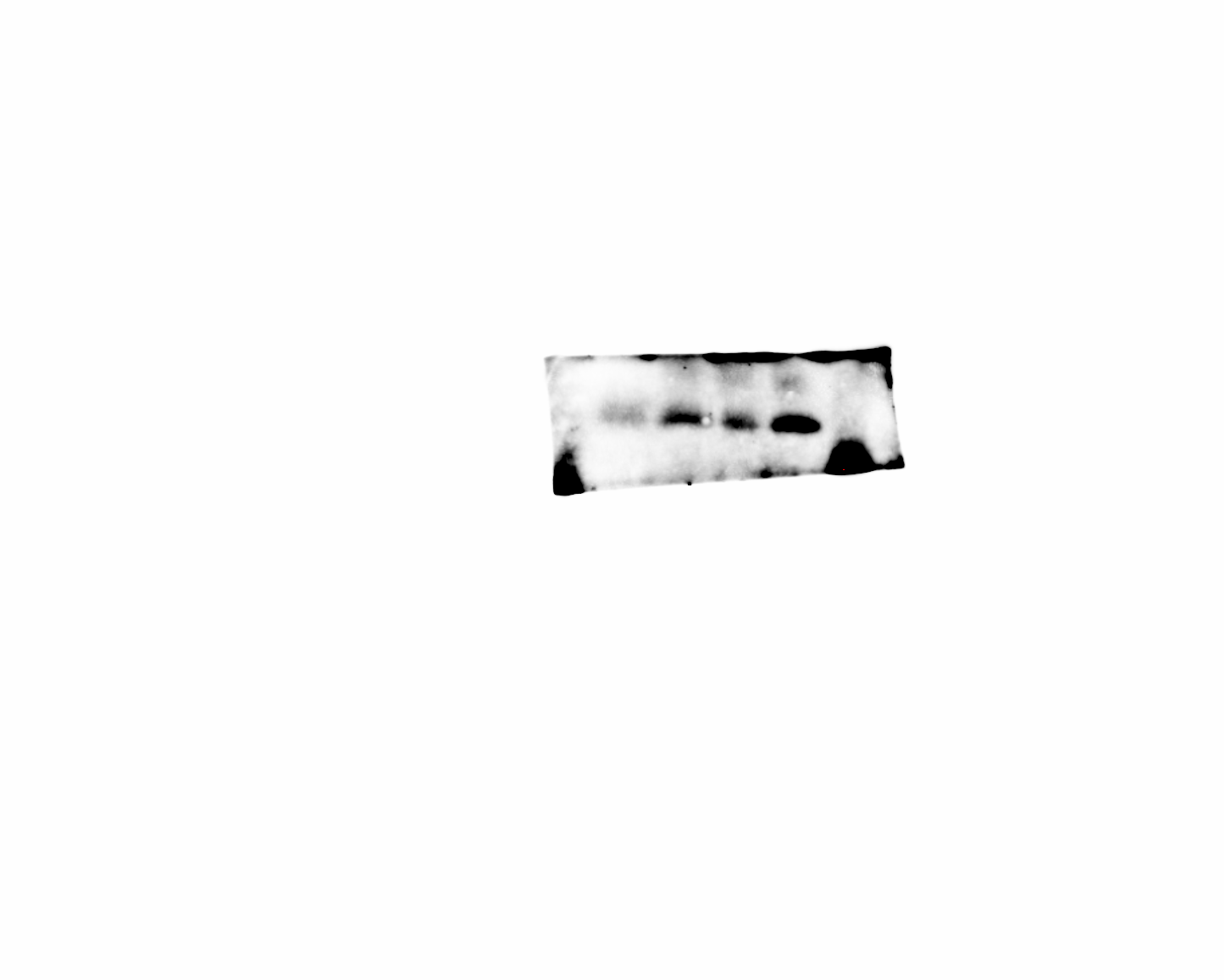

Supplement: Supplementary file 13 — Supplementary Material 13 [file 13098_2024_1389_MOESM13_ESM.tif]

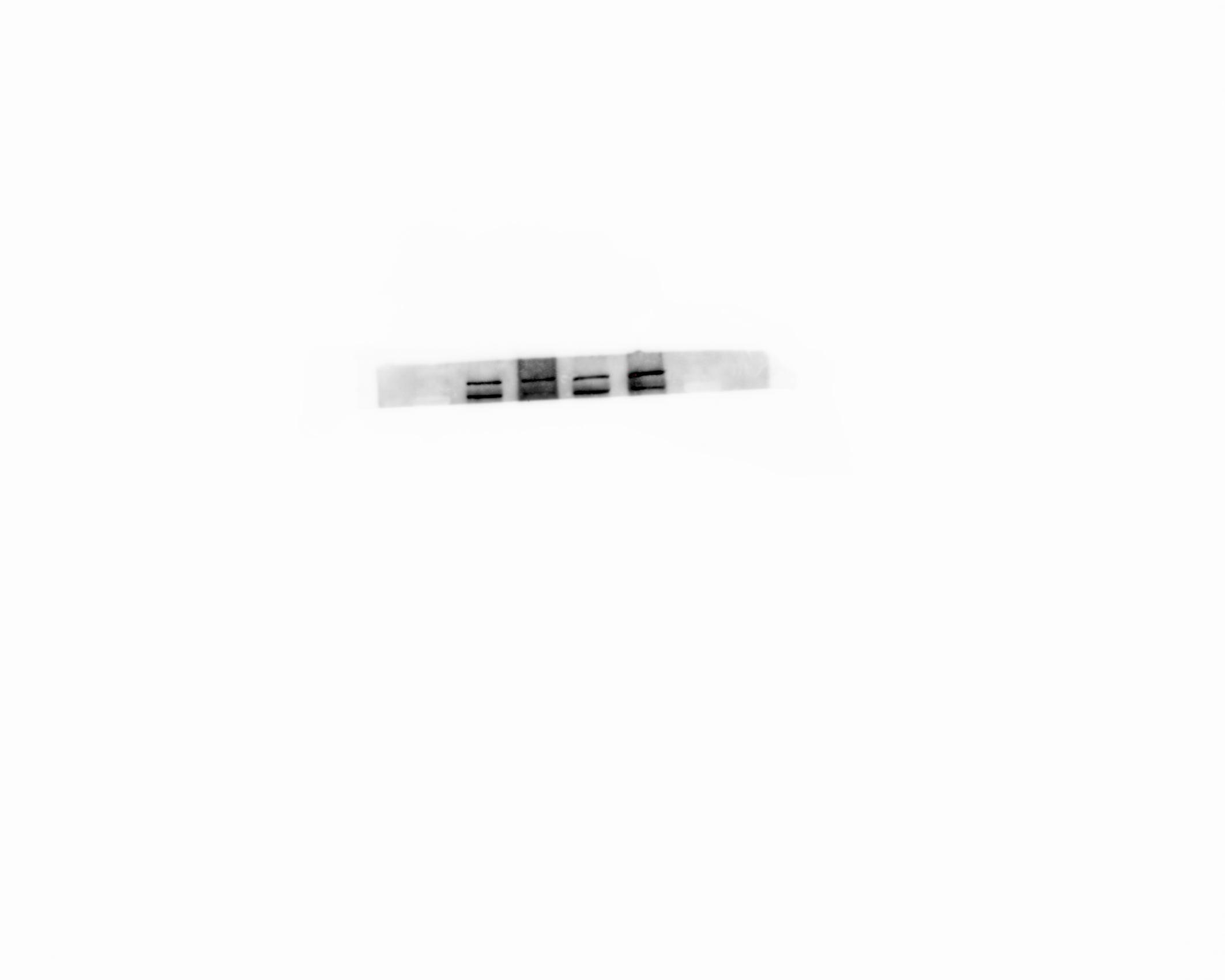

Supplement: Supplementary file 15 — Supplementary Material 15 [file 13098_2024_1389_MOESM15_ESM.tif]
